# FTIP1 Is an Essential Regulator Required for Florigen Transport

**DOI:** 10.1371/journal.pbio.1001313

**Published:** 2012-04-17

**Authors:** Lu Liu, Chang Liu, Xingliang Hou, Wanyan Xi, Lisha Shen, Zhen Tao, Yue Wang, Hao Yu

**Affiliations:** Department of Biological Sciences and Temasek Life Sciences Laboratory, National University of Singapore, Singapore; Cambridge University, United Kingdom

## Abstract

The capacity to respond to day length, photoperiodism, is crucial for flowering plants to adapt to seasonal change. The photoperiodic control of flowering in plants is mediated by a long-distance mobile floral stimulus called florigen that moves from leaves to the shoot apex. Although the proteins encoded by *FLOWERING LOCUS T* (*FT*) in *Arabidopsis* and its orthologs in other plants are identified as the long-sought florigen, whether their transport is a simple diffusion process or under regulation remains elusive. Here we show that an endoplasmic reticulum (ER) membrane protein, FT-INTERACTING PROTEIN 1 (FTIP1), is an essential regulator required for FT protein transport in *Arabidopsis*. Loss of function of *FTIP1* exhibits late flowering under long days, which is partly due to the compromised FT movement to the shoot apex. FTIP1 and FT share similar mRNA expression patterns and subcellular localization, and they interact specifically in phloem companion cells. FTIP1 is required for FT export from companion cells to sieve elements, thus affecting FT transport through the phloem to the SAM. Our results provide a mechanistic understanding of florigen transport, demonstrating that FT moves in a regulated manner and that FTIP1 mediates FT transport to induce flowering.

## Abstract 2

FT-INTERACTING PROTEIN 1 is a novel protein that is involved in transporting florigen, a long-known mobile signal that induces flowering in plants in response to day length, from companion cells to sieve elements in the phloem of Arabidopsis.

## Author Summary

The transition to flowering is the most dramatic phase change in flowering plants and is crucial for reproductive success. Such a transition from vegetative to reproductive growth is controlled by seasonal changes in day length. Studies originally performed in the 1930s were the first to suggest that day length is perceived by a plant's leaves; by contrast, flower formation takes place in the shoot apical meristem (the tip of the shoot that gives rise to plant organs, such as leaves and flowers). The term “florigen” was later proposed to describe a mobile floral stimulus that moves from leaves to the shoot apical meristem to induce flowering. It is only recently that FLOWERING LOCUS T (FT) in *Arabidopsis*, and its orthologs in various other plant species, was identified as being florigen, but how florigen is transported in plants remains completely unknown. Here, we report that a novel ER membrane protein, FT-INTERACTING PROTEIN 1 (FTIP1), interacts with FT in companion cells of the phloem (a specialized type of parenchyma cell in the phloem of the plant's vascular system) and mediates FT protein movement from companion cells to sieve elements (the conducting cells of the phloem), thus affecting FT transport to the shoot apical meristem in *Arabidopsis*. To our knowledge, this study reveals the first regulator that is required for florigen transport and offers new insights into possible florigen transport mechanisms in other flowering plants.

## Introduction

The transition to flowering, which is crucial for the reproductive success, is the most dramatic phase change in flowering plants. Plants are able to adjust the timing of this transition in response to environmental conditions, such as photoperiod, temperature, and availability of nutrients. Classic experiments on the photoperiodic control of flowering in various plants have demonstrated that plant response to day length begins with the perception of photoperiod in leaves, followed by the transmission of a floral stimulus into the shoot apical meristem (SAM), where flowers are generated instead of leaves. Such mobile floral stimulus moving from leaves to the SAM was proposed as “florigen” in the 1930s [1]. Since then, tremendous efforts have been made to understand the molecular nature of this signal. Recent findings have suggested that the proteins encoded by *FLOWERING LOCUS T* (*FT*) in *Arabidopsis* and its orthologs in other plant species are part of the long-sought florigen [2]–[6].

*FT* encodes a member of the phosphatidylethanolamine-binding protein family and acts as a crucial regulator that relays flowering signals from the photoperiod pathway to floral meristem identity genes in *Arabidopsis*, which is a long-day (LD) facultative plant [7]–[10]. Under LDs, *FT* mRNA expression is activated by the CONSTANS (CO) transcriptional regulator in the vascular tissues of leaves and displays circadian rhythm [8],[11]–[14]. It has been suggested that long-distance movement of FT protein from leaves to the shoot apex through the phloem system plays a role in floral induction [2],[4],[5]. In the SAM, FT interacts with the bZIP transcription factor FLOWERING LOCUS D (FD), which in turn activates the downstream floral meristem identity genes such as *APETALA1* (*AP1*) to initiate flower development [8],[9]. Despite the remarkable progress in elucidating FT function, it is so far completely unknown whether and how FT protein transport is regulated. As the abundance of native FT protein is too low to be detectable, it has been hypothesized that simple diffusion of FT protein from companion cells to sieve elements might not be sufficient for transporting FT to the SAM [15].

Here we show that an endoplasmic reticulum (ER) membrane protein, FT-INTERACTING PROTEIN 1 (FTIP1), is required for FT protein transport in *Arabidopsis*. Loss of function of *FTIP1* exhibits late flowering under LDs, which is partly due to the compromised FT movement to the SAM. FTIP1 and FT have similar mRNA expression patterns and subcellular localization, and they interact in vivo in phloem companion cells. Furthermore, FTIP1 is required for FT export from companion cells to sieve elements, thus affecting FT transport through the phloem to the SAM. Our results provide a mechanistic understanding of florigen transport and demonstrate that FT protein moves in a regulated manner and that FTIP1 is involved in mediating the export of FT protein from phloem companion cells to induce flowering.

## Results

### *FTIP1* Regulates Flowering Time under LDs

**Figure 1:**
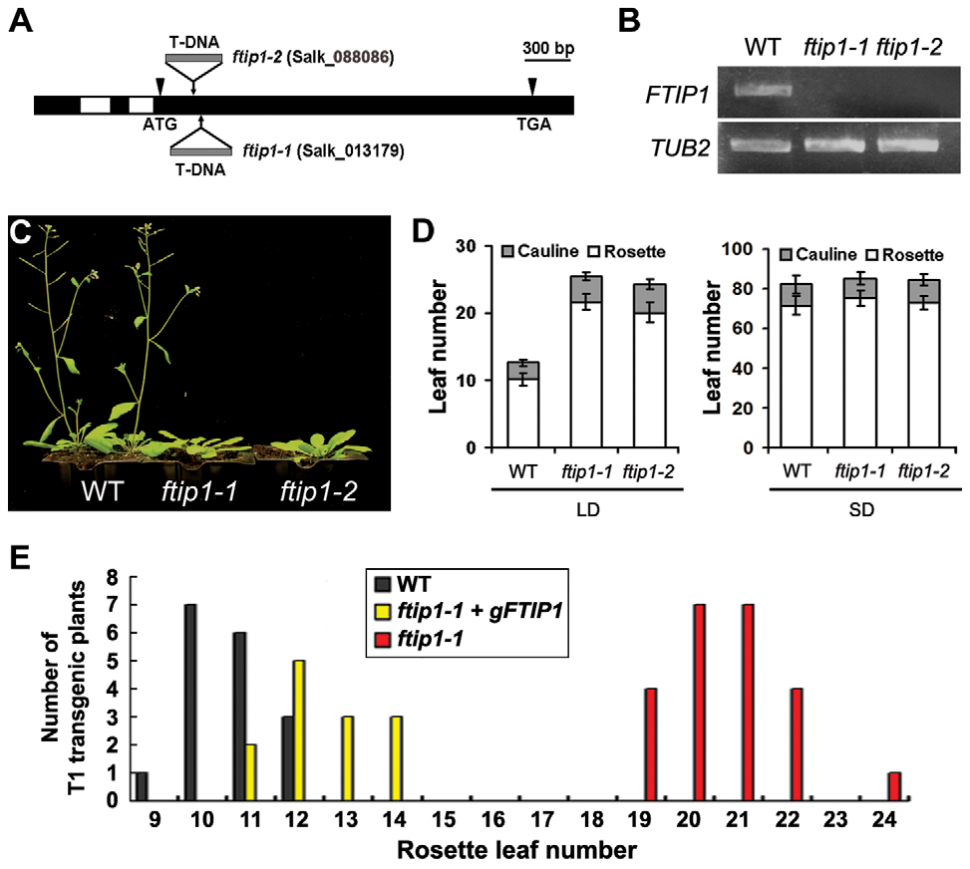
*FTIP1* promotes flowering under LDs. (A) Schematic diagram showing the *FTIP1* coding region and T-DNA insertion mutants. Exons and introns are indicated by black and white boxes, respectively. Two T-DNA insertion lines, *ftip1-1* and *ftip1-2*, were obtained from *Arabidopsis* Biological Resource Center. (B) *FTIP1* expression is undetectable in *ftip1-1* or *ftip1-2* by semi-quantitative PCR using the primers flanking T-DNA insertion sites (Table S2). (C) *ftip1-1* and *ftip1-2* show later flowering than wild-type plants at 35 d after germination under LDs. (D) Flowering time of *ftip1-1* and *ftip1-2* grown under LDs and SDs. Error bars indicate SD. (E) Distribution of flowering time in T1 transgenic plants carrying the *FTIP1* genomic fragment (Figure S2A) in *ftip1-1* background.

To understand how FT function is regulated, we performed yeast two-hybrid screening to identify proteins that interact with FT. Approximately 3 million yeast transformants were screened and 66 colonies were identified on the selective medium (Table S1), among which a partial sequence belonging to an unknown protein with three C2 domains and one phosphoribosyltransferase C-terminal domain (PRT_C) was isolated (Figure S1). The corresponding gene (At5g06850) was therefore named *FT-INTERACTING PROTEIN 1* (*FTIP1*). We isolated two T-DNA insertional alleles, *ftip1-1* (Salk_013179) and *ftip1-2* (Salk_088086), from *Arabidopsis* Biological Resource Center (Figure 1A). The full-length *FTIP1* transcript was undetectable in either homozygous mutant (Figure 1B). Both *ftip1-1* and *ftip1-2* flowered late under LDs, but not under short days (SDs) (Figure 1C,D; Table 1), suggesting that *FTIP1* plays a role in mediating the effect of photoperiod on flowering. We transformed *ftip1-1* with a genomic construct (*gFTIP1*) harboring a 5.1-kb *FTIP1* genomic region including 2.1 kb of the upstream sequence, the 2.4-kb coding sequence, and 0.6 kb of the downstream sequence (Figure S2A). Most *ftip1-1 gFTIP1* T1 transformants exhibited similar or slightly late flowering time as compared to wild-type plants (Figure 1E), demonstrating that *FTIP1* is responsible for promoting flowering particularly under LDs.

**Table 1:** Flowering time of transgenic and mutant plants.

Genotype^a^	Number of Rosette Leaves^b^	Number of Cauline Leaves^b^	n
Experiment 1			
Wild type	9.8±1.0 (8–11)	2.5± 0.5 (2–3)	25
*ftip1-1*	21.8±1.0 (19–24)	4.2±0.7 (3–5)	20
*ftip1-2*	20.2±1.3 (18–23)	4.5±0.7 (3–6)	20
*soc1-2*	24.2±1.3 (22–26)	4.6±0.8 (4–6)	16
*soc1-2 ftip1-1*	40.8±1.5 (39–43)	8.0±0.9 (7–9)	10
*co-1*	18.2±1.3 (16–20)	5.4±0.8 (4–6)	20
*co-1 ftip1-1*	34.7±3.7 (29–40)	7.6±0.5 (7–8)	15
*gi-1*	48.7±4.2 (44–55)	8.6±1.3 (7–11)	14
*gi-1 ftip1-1*	55.3±7.3 (43–64)	10.0±2.2 (8–14)	15
*ft-1*	44.3±6.2 (36–54)	8.8±1.3 (7–11)	16
*ft-1 ftip1-1*	59.2±2.9 (56–64)	11.8±1.9 (9–14)	14
*ft-10*	51.8±3.0 (48–56)	9.8±1.6 (8–12)	15
*ft-10 ftip1-1*	60.8±6.0 (52–67)	11.6±2.0 (9–14)	14
Experiment 2			
Wild type	10.2±1.1 (8–11)	2.3±0.4 (2–3)	20
*ftip1-1*	21.6±1.0 (19–24)	4.1±0.7 (3–5)	20
*ftip1-1 gFTIP1* #3 (T3)	10.4±1.1 (9–12)	2.3±0.7 (2–3)	20
*ftip1-1 gFTIP1* #11 (T3)	10.3±1.1 (8–12)	2.4±0.6 (2–3)	20
*SUC2:FTIP1 ftip1-1*	11.1±1.6 (9–13)	2.4±0.4 (2–3)	15
*FTIP1:4HA:FTIP1 ftip1-1*	11.1±1.8 (9–13)	2.4±0.4 (2–3)	15
*FTIP1:FTIP1:GFP ftip1-1*	12.9±1.3 (11–15)	2.8±0.8 (2–4)	15
*SUC2:FTIP1:GFP ftip1-1*	13.1±1.6 (11–15)	2.4±0.6 (2–4)	15
Experiment 3			
Wild type	9.5±1.0 (8–11)	2.6±0.5 (2–3)	20
*ft-10 tsf-1*	66.7±4.2 (59–70)	10.0±1.4 (8–12)	15
*ft-10 tsf-1 ftip1-1*	67.3±3.5 (62–71)	10.9±1.3 (9–13)	13
Experiment 4			
Wild type	9.5±1.1 (8–11)	2.5±0.6 (2–3)	20
*ftip1-1*	22.5±1.2 (19–24)	3.9±0.9 (3–5)	20
*KNAT1:FT*	6.6±0.8 (5–8)	2.2±0.4 (2–3)	25
*KNAT1:FT ftip1-1*	6.9±1.0 (5–9)	2.3±0.5 (2–3)	25
*KNAT1:FT:GFP*	9.2±0.9 (7–11)	2.5±0.2 (2–3)	25
*KNAT1:FT:GFP ftip1-1*	9.5±1.1 (8–11)	2.7±0.4 (2–3)	25
*SUC2:FT:GFP*	8.7±0.8 (7–10)	2.8±0.6 (2–4)	30
*SUC2:FT:GFP ft-10*	21.5±1.4 (18–25)	4.4±0.7 (3–6)	24
*SUC2:FT:GFP ftip1-1*	12.1±0.8 (11–13)	3.3±0.5 (3–4)	30
*SUC2:FT* c	3.1±0.4 (2–4)	1.7±0.4 (1–2)	30
*SUC2:FT ftip1-1* c	3.7±0.6 (3–5)	1.8±0.5 (1–3)	30
*SUC2:FT ft-10*	5.3±0.7 (4–6)	1.9±0.6 (1–2)	30
*SUC2:GFP:CO* d	4.0±0.4 (3–5)	1.4±0.5 (1–2)	30
*SUC2:GFP:CO ftip1-1* d	4.8±0.4 (4–6)	1.5±0.5 (1–2)	30
Experiment 5			
Wild type	10.2±1.0 (8–11)	2.7±0.5 (2–3)	20
*SUC2:FT:9myc*	7.5±0.6 (6–9)	2.5±0.5 (2–4)	25
*SUC2:FT:9myc ft-10*	20.4±1.3 (18–23)	4.0±0.4 (3–6)	20
*SUC2:FT:9myc ftip1-1*	15.2±0.8 (14–17)	4.3±0.6 (3–5)	25
Experiment 6			
Wild type	10.8±1.2 (8–11)	2.6±0.5 (2–3)	20
*35S:FTIP1* (line 2)	16.2±0.7 (15–18)	3.3±0.5 (3–4)	16
*SUC2:FT:GFP 35S:FTIP1*	12.6±2.2 (10–15)	2.4±0.5 (2–3)	13

a All of the plants are in the same Columbia background and grown under LDs.

b Flowering time is presented as average ± standard deviation (range).

c The flowering time of *SUC2:FT* and *SUC2:FT ftip1-1* is statistically different (*p* = 7.2×10^−4^).

d The flowering time of *SUC2:GFP:CO* and *SUC2:GFP:CO ftip1-1* is statistically different (*p* = 4.1×10^−11^). Statistical analyses were performed using a two-tailed unpaired Student's *t* test.

### Gene Expression and Subcellular Localization of FTIP1

**Figure 2:**
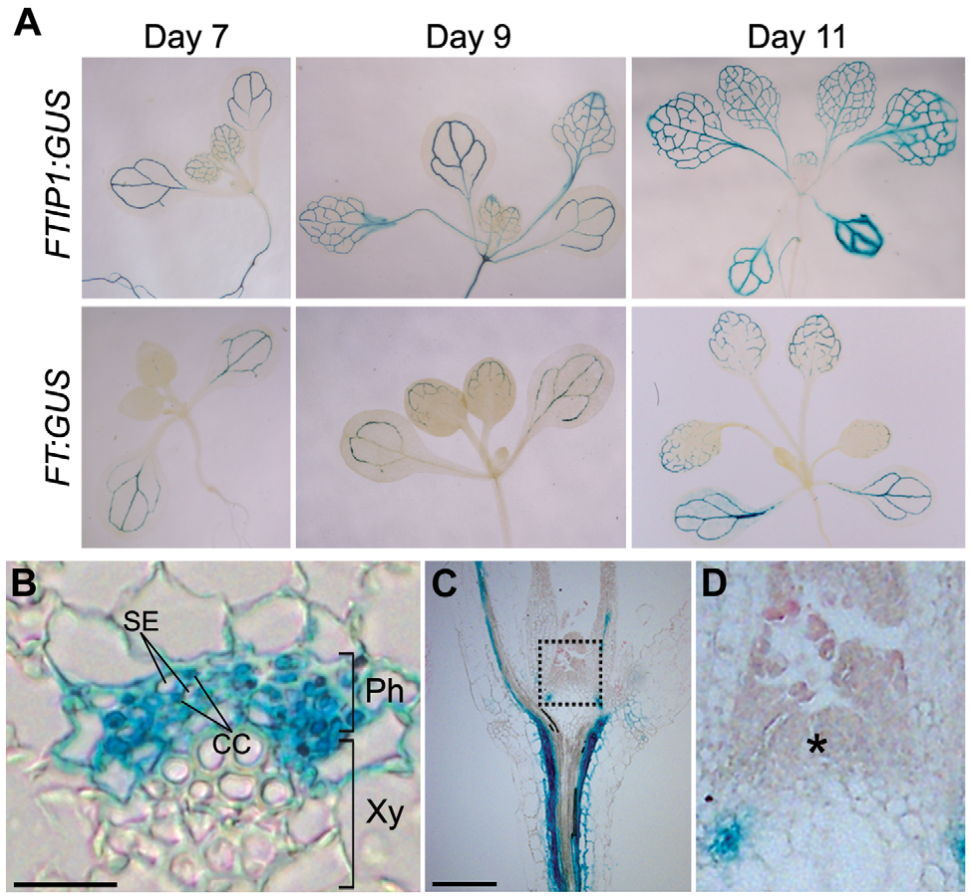
*FTIP1* is expressed in vascular tissues. (A) Comparison of GUS staining of *FTIP1:GUS* and *FT:GUS* grown under LDs for 7 to 11 d. (B) Cross-section of the primary vein of the first rosette leaf from an 11-d-old *FTIP1:GUS* seedling. Ph, phloem; Xy, xylem; SE, sieve element; CC, companion cell. Bar, 50 µm. (C) Longitudinal section through an 11-d-old *FTIP1:GUS* seedling. Bar, 100 µm. (D) A higher magnification of the area within the box indicated in (C). Asterisk indicates the shoot apical meristem.

**Figure 3:**
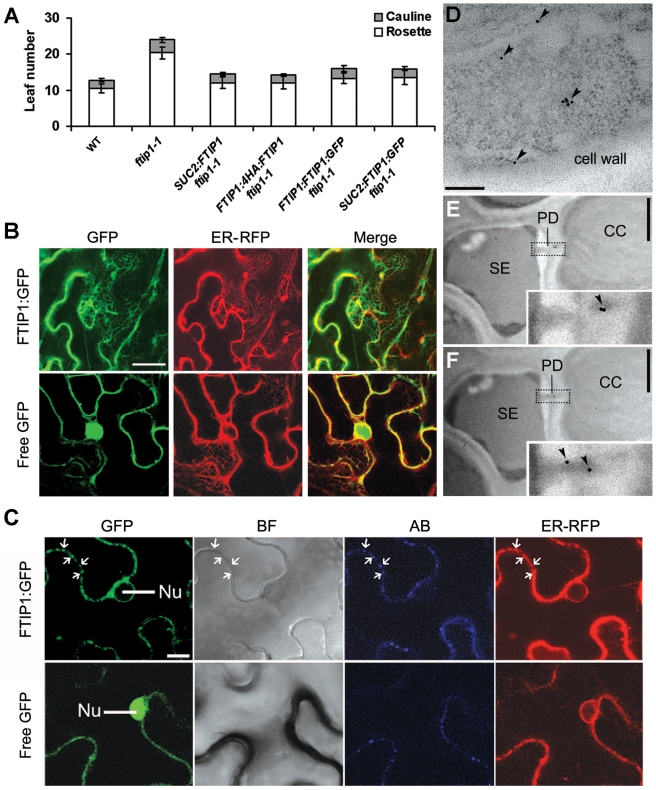
Subcellular localization of FTIP1. (A) Flowering time of various transgenic plants grown under LDs. Error bars indicate SD. (B and C) Subcellular localization of FTIP1:GFP and free GFP in *N. benthamiana* leaf epidermal cells. (B) As compared to free GFP, FTIP1:GFP is mostly colocalized with an ER marker. (C) Both FTIP1:GFP and callose are enriched in the same regions at the cell wall (arrows). GFP, GFP fluorescence; ER-RFP, RFP fluorescence of an ER marker [17]; Merge, merge of GFP and RFP; BF, bright field image; AB, aniline blue staining; Nu, nucleus. Bars: (B), 20 µm; (C), 10 µm. (D–F) Analysis of 4HA:FTIP1 localization in CC-SE complexes in the first rosette leaves of 15-d-old *FTIP1:4HA:FTIP1 ftip1-1* by immunogold electron microscopy. (D) 4HA:FTIP1 is localized in the phloem companion cell. Arrowheads indicate the locations of gold particles. (E,F) 4HA:FTIP1 is localized in the plasmodesma that connects a CC with a SE in two continuous sections. Arrowheads in insets show the location of gold particles in enlarged PD regions. SE, sieve element; CC, companion cell; PD, plasmodesma. Bars: (D), 250 nm; (E and F), 1 µm.

We tested *FTIP1* expression in various tissues of wild-type plants using quantitative real-time PCR and found its highest expression in leaves and stems (Figure S3). To examine the detailed expression pattern of *FTIP1*, we generated a *FTIP1:β-glucuronidase (GUS)* reporter construct in which the same 2.1-kb *FTIP1* upstream sequence included in *gFTIP1* for the gene complementation test was fused to the *GUS* reporter gene (Figure S2A). We created 23 independent *FTIP1:GUS* lines, most of which showed similar GUS staining patterns. A representative line was selected to monitor the detailed expression pattern of *FTIP1*. *FTIP1:GUS* showed specific GUS staining in vascular tissues of various plant organs (Figure S2B–H). Notably, in developing seedlings during the floral transition occurring 7 d after germination, *FTIP1:GUS* and *FT:GUS* [14] shared similar GUS staining patterns in vascular tissues of cotyledons and rosette leaves, although the former had a relatively broad and intensive staining pattern (Figure 2A). A cross-section of a primary leaf vein revealed that *FTIP1:GUS* expression was specifically located in the phloem including companion cells (Figure 2B), which is similar to the *FT:GUS* expression pattern [14]. Neither *FTIP1:GUS* nor *FT:GUS* was expressed in the SAM (Figure 2C,D; Figure S2C) [14]. Furthermore, the late-flowering phenotype of *ftip1-1* was rescued by the expression of *FTIP1* coding sequence driven by the promoter of *SUCROSE TRANSPORTER 2* (*SUC2*) (Figure 3A), which is active specifically in phloem companion cells [16]. These results suggest that *FTIP1* functions in the phloem to promote flowering.

Given that *FTIP1* functions in flowering time control, we investigated whether its expression is regulated by known flowering genetic pathways. *FTIP1* expression was not regulated by photoperiod and did not exhibit an obvious circadian rhythm under LDs (Figure S4A,E). Similarly, vernalization treatment did not affect *FTIP1* expression (Figure S4B), and GA treatment did not affect *FTIP1* expression and the flowering phenotype of *ftip1-1* (Figure S4C,D). In addition, *FTIP1* expression was also not altered in several mutants tested in known flowering genetic pathways (Figure S5). These observations imply that flowering signals may not regulate *FTIP1* function through affecting its mRNA levels.

Next, we examined the subcellular localization of FTIP1 through monitoring the signal of the green fluorescent protein (GFP) fused with FTIP1 under the control of *FTIP1* or *SUC2* promoter, respectively. Both constructs could rescue the late flowering phenotype of *ftip1-1* (Figure 3A). However, we could not detect fluorescent signal from either *SUC2:FTIP1:GFP ftip1-1* or *FTIP1:FTIP1:GFP ftip1-1* transgenic lines, indicating that FTIP1 protein might be present at very low abundance in plant cells. Alternatively, we transiently expressed *35S:FTIP1:GFP* with various fluorescent protein-tagged organelle markers in *N. benthamiana* leaf epidermal cells and found that FTIP1:GFP was mostly colocalized with an endoplasmic reticulum (ER) marker (Figure 3B,C; Figure S6) [17]. We did not observe FTIP1:GFP signals in the nucleus (Figure 3C). Notably, at the cell wall, FTIP1:GFP colocalized with callose deposition stained with aniline blue, which marks the position of plasmodesmata (Figure 3C).

To precisely localize FTIP1, we performed immunoelectron microscopy on an *FTIP1:4HA:FTIP1 ftip1-1* transgenic line, in which *FTIP1:4HA:FTIP1* was able to rescue the flowering defect of *ftip1-1* (Figure 3A). The result revealed that 4HA:FTIP1 was specifically localized in phloem companion cells (Figure 3D) and plasmodesmata between companion cells and sieve elements (Figure 3E,F; Figure S7), where the ER membrane runs through.

### FTIP1 Interacts with FT in Phloem Companion Cells

**Figure 4:**
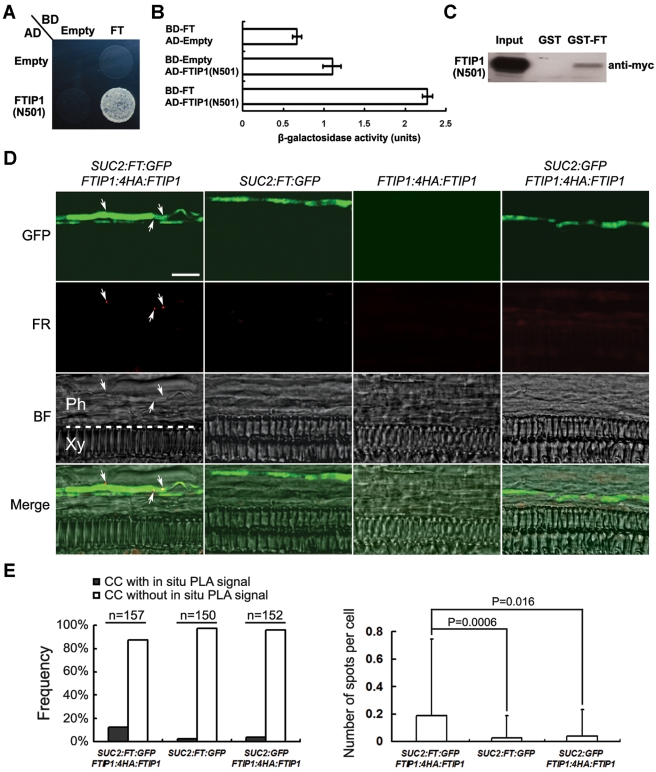
FTIP1 interacts with FT. (A) Yeast two-hybrid assay of interaction between FT and the N-terminal region of FTIP1 (aa 1–501; N501), which contains three C2 domains (Figure S1A). Yeast cells were grown on SD-His/-Trp/-Leu medium supplemented with 30 mM 3-amino-1, 2, 4-triazole. (B) Quantification of the interaction between FT and FTIP1 (N501) in yeast by β-galactosidase assays. (C) In vitro pull-down assay of interaction between FT and FTIP1 (N501). “Input” indicates 5% of myc-labeled FTIP1 (N501) subjected to pull-down by GST and GST-FT. (D) In situ PLA detection of interaction between FT:GFP and 4HA:FTIP1 in phloem companion cells of an 11-d-old *Arabidopsis* leaf. Protein-protein interactions are visualized as small red spots indicated by arrows. The dotted line indicates the border between phloem and xylem. GFP, GFP fluorescence; FR, far red fluorescence; BF, bright field image; Merge, merge of GFP, FR, and BF; Ph, phloem; Xy, xylem. Bar, 10 µm. (E) Quantification of in situ PLA data. Statistical analysis was performed by counting the number of far red fluorescence signals (red spots) in the phloem companion cells that could be identified with the GFP signal. The left panel shows the frequency histogram of appearance of red spots found in phloem companion cells. The number of sections examined for each genotype is listed above the histogram. The right panel shows the average number of red spots per phloem companion cell. Statistical analysis was performed using a two-tailed unpaired Student's *t* test. The results are considered statistically significant at *p*<0.05.

Several pieces of evidence, including the initial identification of FTIP1 as an FT interacting partner, similar tissue expression pattern of *FTIP1* and *FT*, and similar late-flowering phenotype exhibited by *ftip1* and *ft* mutants specifically under long days, point to a possible role of FTIP1 in mediating FT function in the control of flowering time. Thus, we further carried out a detailed analysis of the interaction between FTIP1 and FT. As revealed in our yeast two-hybrid screening, a truncated FTIP1 protein devoid of the PRT_C domain interacted with FT in both yeast two-hybrid and GST pull-down assays (Figure 4A–C), whereas no interaction was detected using the full-length FTIP1 (unpublished data). Since the PRT_C domain of FTIP1 was predicted to be a membrane-targeted domain according to a protein topology analysis (Figure S1), the full-length FTIP1 protein might not be in the membrane-bound state in yeast cells or under in vitro conditions and thus might undergo inappropriate folding, which prevents its interaction with FT. Alternatively, in yeast two-hybrid assay the full-length FTIP1 protein might be membrane-bound and unable to reconstitute a functional transcription factor in the yeast nucleus to drive the reporter gene expression.

We transiently expressed *35S:FTIP1:GFP* with *35S:FT:RFP* in *N. benthamiana* leaf epidermal cells and revealed that both FTIP1:GFP and FT:RFP were colocalized to ER connected to the nuclear envelope (Figure S8). However, in contrast to FTIP1:GFP, FT:RFP was also localized in the nucleus, which is consistent with a previous observation [9]. These results indicate that FTIP1 may not directly mediate FT function in transcriptional regulation of other target genes.

To test whether and how FT interacts with FTIP1 in vivo, we performed in situ Proximity Ligation Assay (PLA) [18], in which dual recognition of target proteins by pairs of affinity probes generates an amplifiable DNA reporter molecule that serves as a surrogate marker for interacting proteins, to examine the subcellular localization of FT and FTIP1 interaction at single-molecule resolution in the leaves of 11-d-old *SUC2:FT:GFP; FTIP1:4HA:FTIP1* transgenic plants. PLA signals visualized as small red dots were specifically detected in the phloem companion cells of *SUC2:FT:GFP; FTIP1:4HA:FTIP1*, but barely in those transgenic plants containing only *SUC2:FT:GFP*, *FTIP1:4HA:FTIP1*, or *SUC2:GFP; FTIP1:4HA:FTIP1* (Figure 4D,E). This result demonstrates that FT and FTIP1 physically interact in close proximity in phloem companion cells.

### FTIP1 Controls the Export of FT Protein from Phloem Companion Cells to Sieve Elements

**Figure 5:**
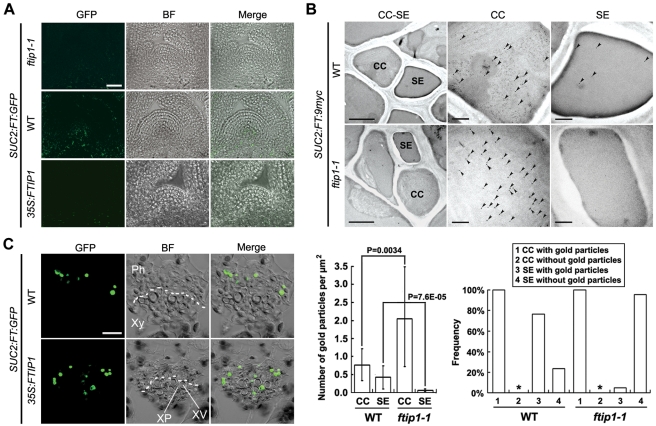
FTIP1 is required for FT protein transport. (A) Confocal analysis of FT:GFP protein distribution in the apical region of 11-d-old *SUC2:FT:GFP* seedlings in different genetic backgrounds. Bar, 20 µm. (B) Analysis of FT:9myc distribution in CC-SE complexes in the first rosette leaves of 15-d-old *SUC2:FT:9myc* and *SUC2:FT:9myc ftip1-1* seedlings by immunogold electron microscopy using anti-myc antibody. The upper left panels show the representative CC-SE complexes, while higher magnification views of CCs or SEs are shown in the upper middle or right panels, respectively. Arrowheads indicate the locations of gold particles. The lower left panel shows the quantification of FT:9myc immunogold signals in CCs and SEs of *SUC2:FT:9myc* (WT background) or *SUC2:FT:9myc ftip1-1* (*ftip1-1* background). The data are presented as the mean number of gold particles per µm^2^ plus or minus standard deviation. Statistical analysis was performed using a two-tailed unpaired Student's *t* test. The results are considered statistically significant at *p*<0.05. The lower right panel shows the frequency histogram of appearance of FT:9myc immunogold signals in CCs and SEs in all examined sections of *SUC2:FT:9myc* or *SUC2:FT:9myc ftip1-1*. Asterisks indicate that in all sections examined, the frequency we observed CCs without gold particles is zero. Bars: upper left panels, 2 µm; upper middle and right panels, 0.5 µm. (C) Confocal analysis of FT:GFP protein distribution in the primary vein of the first rosette leaves from 11-d-old *SUC2:FT:GFP* seedlings in different genetic backgrounds. The dotted lines indicate the approximate boarder between xylem and phloem. GFP, GFP fluorescence; BF, bright field image; Merge, merge of GFP and BF; CC, companion cell; SE, sieve element; Ph, phloem; Xy, xylem; XP, xylem parenchyma; XV, xylem vessel. Bar, 20 µm.

**Figure 6:**
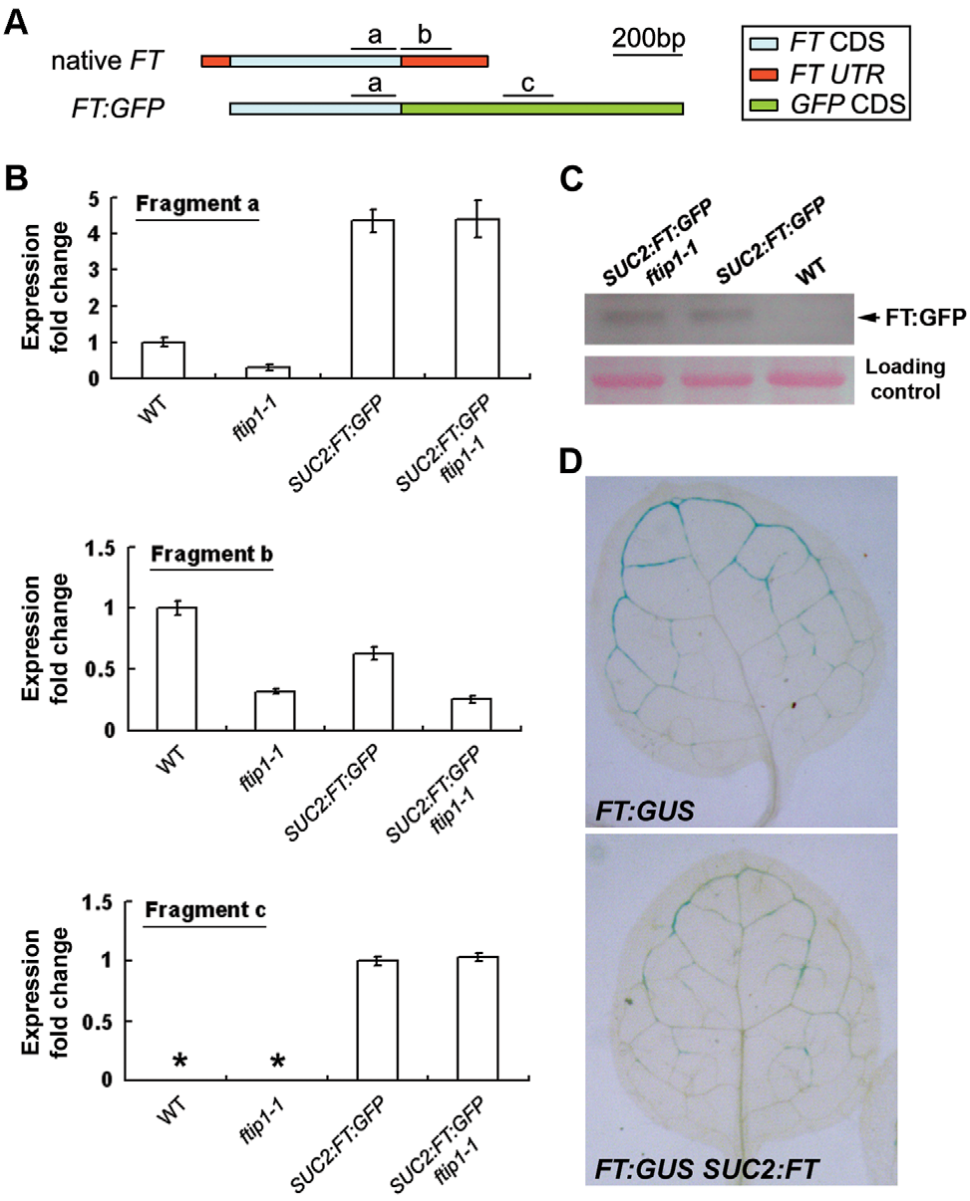
FTIP1 does not regulate *FT* mRNA or protein stability. (A) Schematic diagrams showing native *FT* and transgenic *SUC2:FT:GFP* transcripts. The fragments labeled with a, b, and c indicate amplicons in real-time PCR analyses shown in (***B***). Fragments a, b, and c were amplified with primers FT-F and FT-R, FT(UTR)-F and FT(UTR)-R, and GFP-F1 and GFP-R1 (Table S1), respectively. (B) Examination of steady-state levels of *FT* or *FT:GFP* mRNA in wild-type and *ftip1-1* backgrounds. Amplification of fragment a, which detects the amplicon in both native *FT* and transgenic *FT:GFP* transcripts, shows that native *FT* expression is downregulated in *ftip1-1* versus wild-type, whereas total *FT* expression (including minor native *FT* and major transgenic *FT:GFP* expression) remains unchanged in *SUC2:FT:GFP ftip1-1* versus *SUC2:FT:GFP*. Although the former indicates that FTIP1 affects the steady-state levels of native *FT* expression, the latter implies that FTIP1 does not directly affect *FT* mRNA stability. Amplification of fragment c, which only detects the amplicon in transgenic *FT:GFP* transcripts, further supports that FTIP1 does not directly affect *FT* mRNA stability as transgenic *FT:GFP* expression is not changed in *ftip1-1*. Amplification of fragment b, which only detects the amplicon in native *FT* transcripts, shows that native *FT* expression is also downregulated in *SUC2:FT:GFP* transgenic plants. 9-d-old seedlings grown under LDs were harvested for expression analysis by quantitative real-time PCR. Results were normalized against the expression of *TUB2*. Asterisks indicate that the expression of fragment c was undetectable in wild-type and *ftip1-1* seedling. Error bars indicate SD. (C) Western blot analysis using anti-GFP antibody shows the comparable abundance of FT:GFP protein in wild-type and *ftip1-1* plants. Ponceau S staining of the membrane is used as a loading control. (D) GUS staining of rosette leaves of 9-d-old *FT:GUS* and *FT:GUS SUC2:FT* seedling.

The findings on the interaction between FT and FTIP1, and FTIP1 localization to ER and plasmodesmata prompted us to hypothesize that FTIP1 may regulate FT export from phloem companion cells. To this end, we first examined whether FTIP1 affects FT transport to the SAM during the floral transition. We generated a *SUC2:FT:GFP* transgenic line as previously described [2]. As this transgenic allele could significantly rescue the late-flowering phenotype of the *FT* null mutant, *ft-10* (Table 1), we further crossed this *SUC2:FT:GFP* allele with *ftip1-1* and *35S:FTIP1*. Confocal analysis of the distribution of FT:GFP fusion protein revealed that in 11-d-old seedlings, which were undergoing the floral transition, FT:GFP was clearly detected in the inner cone-like region of the SAM in wild-type background, but not in *ftip1-1* (Figure 5A). In contrast, the distribution of free GFP protein was comparably undetectable in the inner region of the SAM in wild-type and *ftip1-1* (Figure S9A), indicating a specific effect of FTIP1 on FT:GFP distribution in the SAM during the floral transition. In agreement with the above observations, *SUC2:FT:GFP ftip1-1* flowered later than *SUC2:FT:GFP* (Table 1). Since the abundance of FT:GFP mRNA and protein in *SUC2:FT:GFP* was not downregulated in *ftip1-1* (Figure 6A–C), the difference in FT:GFP distribution in the SAM between wild-type and *ftip1-1* plants suggests a role of FTIP1 in regulating FT transport rather than *FT* mRNA or protein abundance.

As *FTIP1* was expressed in the phloem (Figure 2) and its protein was localized in phloem companion cells (Figure 3D–F; Figure 4D), we examined whether FTIP1 affects FT transport from companion cells to sieve elements in a newly created *SUC2:FT:9myc* line in wild-type and *ftip1-1* backgrounds using immunoelectron microscopy (Figure 5B). This *SUC2:FT:9myc* transgenic allele substantially rescued the late-flowering phenotype of *ft-10* (Table 1), indicating that FT:9myc retains the biological function of endogenous FT protein. Signals corresponding to FT:9myc could be specifically detected by anti-myc antibody in the phloem of the transgenic plants harboring *SUC2:FT:9myc* (Figure 5B; Figure S10). Quantitative analysis of labeling density of FT:9myc in companion cell-sieve element complexes showed that although all sections from *SUC2:FT:9myc* and *SUC2:FT:9myc ftip1-1* displayed FT:9myc labeling in companion cells (Figure 5B, lower right panel), there was an approximate 3-fold enrichment of labeling density in *ftip1-1* over wild-type background (Figure 5B, lower left panel). More importantly, we detected FT:9myc labeling in sieve elements in nearly 80% of the wild-type sections, whereas only 4% of *ftip1-1* sections displayed FT:9myc labeling in sieve elements (Figure 5B, lower right panel). In addition, the labeling density of FT:9myc in sieve elements was much higher in wild-type than in *ftip1-1* (Figure 5B, lower left panel). Thus, in the absence of FTIP1, FT:9myc accumulated in companion cells and its transport to sieve elements was compromised. In agreement with this result, *SUC2:FT:9myc ftip1-1* displayed later flowering than *SUC2:FT:9myc* (Table 1). As *ftip1-1* also delayed flowering in *SUC2:FT* and *SUC2:GFP:CO* where *CO* directly promotes the endogenous *FT* expression (Table 1) [12], it seems that FTIP1 similarly affects the promotive effect of untagged FT protein on flowering as other FT fusion proteins used in this study. These observations support that FTIP1 regulates FT export from phloem companion cells to sieve elements, thus affecting FT transport through the phloem to the SAM. Consistent with this conclusion, the early-flowering phenotype caused by expression of *FT* or *FT:GFP* under the control of the *KNAT1* promoter, which is active in the SAM [12], was not affected by *ftip1-1* (Table 1).

Unlike other flowering promoters, overexpression of *FTIP1* surprisingly caused late flowering (Figure S11; Table 1). Confocal analysis showed that the expression of FT:GFP protein in the inner region of the SAMs of 11-d-old seedlings was substantially lower in *35S:FTIP1* than in wild-type plants (Figure 5A; Figure S9A). In the primary leaf vein, FT:GFP driven by the *SUC2* promoter was exclusively detected in phloem companion cells in wild-type background, whereas in *35S:FTIP1*, the distribution of FT:GFP signals was detected in both phloem companion cells and xylem parenchyma cells (Figure 5C). However, the free GFP driven by the *SUC2* promoter remained in phloem companion cells of *35S:FTIP1* as compared to wild-type plants (Figure S9B). These observations demonstrate that that ectopic expression of *FTIP1* specifically deregulates the transport of FT:GFP protein out of the phloem system, an effect previously shown for a viral movement protein [19]. This could compromise the eventual distribution of FT:GFP in the SAM of *35S:FTIP1* and thus delay flowering.

### *FTIP1* Is Involved in Feedback Regulation of *FT* mRNA Expression

During the floral transition, FT interacts with FD in the SAM to promote the expression of *AP1* and other flowering genes such as *SUPPRESSOR OF OVEREXPRESSION OF CONSTANS 1* (*SOC1*) [8],[9],[20]. As expected, the expression of these genes was downregulated in *ftip1-1* in which FT transport is defective (Figure S12A). Surprisingly, *FT* expression was also downregulated in *ftip1-1*, whereas the expression of *CO*, a direct upstream activator of *FT*, was not significantly changed (Figure S12). As FTIP1 protein is not localized in the nucleus, it is unlikely that FTIP1 directly controls *FT* transcription. To address whether FTIP1 could regulate the stability of *FT* transcripts, we compared the levels of *FT* transcripts generated from the native and *SUC2:FT:GFP* transgenic loci. Although steady-state levels of native *FT* expression were downregulated in *ftip1-1*, total *FT* expression including native *FT* and transgenic *FT:GFP* expression remained unchanged in *SUC2:FT:GFP ftip1-1* (Figure 6A,B). In addition, the abundance of FT:GFP fusion protein remained unchanged in wild-type and *ftip1-1* backgrounds (Figure 6C). These results suggest that *FTIP1* may not be directly involved in regulating *FT* mRNA or protein stability. Meanwhile, we observed downregulation of native *FT* expression in *SUC2:FT:GFP* (Figure 6A,B) and reduced *FT:GUS* staining in *SUC2:FT* (Figure 6D). These results are in agreement with the observation in a previous study [2] implying that an excessive accumulation of FT protein in phloem companion cells caused by the *SUC2* promoter might directly or indirectly result in a reduction in native *FT* mRNA expression through a negative feedback loop. This may explain the observed downregulation of native *FT* expression in *ftip1-1*, where defective export of FT protein causes accumulation of FT protein in phloem companion cells (Figure 5B).

## Discussion

Our results have demonstrated that FTIP1 and FT share similar mRNA expression patterns and subcellular localization, and that they interact in vivo in phloem companion cells. During the floral transition, the FT:GFP accumulation at the SAM is compromised in *ftip1* mutants, which eventually exhibit late flowering under LDs. Consistently, FTIP1 is required for FT:9myc export from phloem companion cells to sieve elements, thus affecting the flowering time of *SUC2:FT:9myc*. In addition, overexpression of *FTIP1* causes the transport of FT:GFP out of the phloem system, which also results in late flowering. These observations suggest that FT protein moves from phloem companion cells to sieve elements in a regulated manner and that a subtle regulation of FTIP1 activity is indispensable for the export of FT protein from phloem companion cells to induce flowering.

We envisage that in addition to FTIP1 and FT, florigen transport should involve other relevant regulators. First, although the transport of FT:9myc protein from phloem companion cells to sieve elements in *ftip1-1* is significantly compromised, it is not completely abolished. This implies either that there is a basal level of diffusion of FT protein or that FT transport also depends on other regulators that share a redundant function with FTIP1 in mediating FT export from phloem companion cells. Furthermore, previous examinations of the spatial distribution of FT:GFP fusion protein in both *Arabidopsis* and rice have shown that FT:GFP accumulates in the rib zone beneath the SAM in a conical shape [2],[3], indicating that the movement of FT protein from phloem to the rib zone is not a simple diffusion process. As *FTIP1* is clearly not expressed in the whole SAM (Figure 2C,D), regulation of FT protein transport from the phloem stream to the rib zone might also involve other regulators. The requirement of other regulators for FT protein transport is supported by the genetic analysis showing that *ft* mutants display much later flowering than *ftip1* (Table 1). Potential candidates for these regulators include *FTIP1* homologs (Figure S13A) because some combinations of *ftip1* with loss-of-function mutants of *FTIP1* homologs show much later flowering than any single mutant (unpublished data).

Second, the late-flowering phenotype of *ft* mutants is further enhanced by *ftip1-1* (Table 1), indicating that *FTIP1* may be required for transporting other flowering molecules in addition to FT. A potential candidate could be *TWIN SISTER OF FT* (*TSF*), which encodes another phosphatidylethanolamine-binding protein with very high sequence similarity with FT [21],[22]. Mutation of *TSF* further enhances the late flowering of *ft* mutants, and the resulting double mutants fail to accelerate flowering in response to LD conditions [21],[22]. The expression domain of *TSF* also overlaps with that of *FTIP1* [21]. Furthermore, loss of function of *FTIP1* does not further delay flowering of *ft-10 tsf-1* under LD conditions (Table 1). These data support that TSF functions redundantly with FT and could be another molecule whose transport is affected by *FTIP1*.

As both FTIP1 and FT proteins are localized to ER, regulation of FT movement by FTIP1 across the border between companion cells and sieve elements might be partly mediated by a continuous ER network within plasmodesmata [23],[24]. In plasmodesmata, the ER becomes appressed to form the central axial desmotubule surrounded by the plasma membrane continuum between adjacent cells [25]. Although it has been suggested that the desmotubule is not the main route for plasmodesmatal transport, some molecules are known to be transported through this channel [26]. In contrast, the space between the desmotubule and the plasma membrane, which is referred as the cytoplasmic sleeve, is the proposed place where the general trafficking of molecules and ions occurs [25]. Because FTIP1 possesses a membrane-targeted PRT_C domain (Figure S1) and is localized to plasmodesmata (Figure 3C,E,F), it is likely that the C-terminus of FTIP1 is anchored to the desmotubule. How FTIP1 is oriented in plasmodesmata is an important question as its N-terminus, which is included in the region that interacts with FT protein (Figure 4A–C), might face either the cytoplasmic sleeve or the interior of the desmotubule. Further addressing this question will help to identify the route where FT protein passes through plasmodesmata and other possible factors involved in FT transport. Based on the size of FT:GFP, the route through the cytoplasmic sleeve might be possible for FT transport as the current understanding is that molecules larger than 27 kDa do not move readily through desmotubule [23].

The presence of C2 domains and a transmembrane domain in FTIP1 and its close homologs in *Arabidopsis* makes them topologically resemble synaptotagmins (Figure S13A) that constitute a family of membrane-trafficking proteins widely found in plants and animals. In *Arabidopsis*, the synaptotagmin SYTA has been shown to regulate endosome recycling and movement protein-mediated trafficking of plant virus genomes through plasmodesmata [27]. Our finding on the function of FTIP1 in mediating FT export from phloem companion cells to sieve elements, together with the proposed SYTA function, implies that synaptotagmin-like proteins may serve as essential regulators that mediate the transport of macromolecules in plants. Another *FTIP1*-like gene, *QUIRKY* (*QKY*; At1g74720), has been suggested to contribute to plant organ organogenesis mediated by the receptor-like kinase *STRUBBELIG* [28], implying a role of *QKY* in intercellular signaling. As FTIP1-like proteins are highly conserved in the angiosperms (Figure S13B), further investigation of *FTIP1* and its homologs might shed light on the conserved mechanisms underlying which flowering plants regulate cell-to-cell communication to coordinate the growth and development.

## Materials and Methods

### Plant Materials

*Arabidopsis* plants were grown at 22°C under long days (16 h light/8 h dark) or short days (8 h light/16 h dark). The mutants *ftip1-1*, *ftip1-2*, *co-1*, *gi-1*, *ft-1* (L*er ft-1* introgressed into Col), *ft-10*, *tsf-1*, *soc1-2*, *agl24-1*, *fve-3*, and *svp-41* are in Columbia (Col) background, while *co-2*, *fca-1*, *fpa-1*, and *fve-1* are in Landsberg *erecta* (L*er*) background.

### Plasmid Construction and Plant Transformation

To construct *35S:FTIP1*, the cDNA encoding *FTIP1* was amplified with primers and cloned into pGreen-35S [29]. For the complementation test, a 5.1-kb *FTIP1* genomic fragment (*gFTIP1*) was amplified and cloned into pHY105 [28]. Based on this construct, *FTIP1:FTIP1:GFP* and *FTIP1:4HA:FTIP1* were generated using a modified QuikChange Site-Directed Mutagenesis approach [30]. The cDNAs encoding GFP and 4HA were amplified. The resulting PCR fragments were annealed to the methylated template plasmid DNA containing *gFTIP1* and elongated with the *PfuTurbo* DNA polymerase (Stratagene). Upon *Dpn*I digestion, the mutated plasmids containing either *GFP* or *4HA* were recovered from *E. coli* transformation. To construct pGreen-SUC2 and pGreen-KNAT1, *SUC2* and *KNAT1* promoters were amplified from Col genomic DNA and cloned into pHY105 [28]. To construct *SUC2:FTIP1*, the cDNA encoding FTIP1 was amplified and cloned into pGreen-SUC2. Based on *SUC2:FTIP1* and *35S:FTIP1*, *SUC2:FTIP1:GFP* and *35S:FTIP1:GFP* were generated using the same modified QuikChange Site-Directed Mutagenesis approach [30] for creating *FTIP1:FTIP1:GFP*. To construct *SUC2:FT* and *KNAT1:FT*, the cDNA encoding FT was amplified and cloned into pGreen-SUC2 and pGreen-KNAT1, respectively. Based on the constructs of *SUC2:FT* and *KNAT1:FT*, *SUC2:FT:GFP, SUC2:FT:9myc* and *KNAT1:FT:GFP* were generated using the same modified QuikChange Site-Directed Mutagenesis approach [30] for creating *FTIP1:FTIP1:GFP*. To construct *35S:FT:RFP*, the cDNA encoding RFP was amplified and cloned into pGreen-35S to generate pGreen-35S-RFP. The cDNA encoding FT was subsequently amplified and cloned into pGreen-35S-RFP. To construct *FTIP1:GUS*, the 2.1-kb *FTIP1* 5′ upstream sequence was amplified and cloned into pHY107 [29]. All transgenic plants were created using the floral dip method [31] and screened by Basta on soil.

### Yeast Two-Hybrid Assay

All vectors used in yeast two-hybrid assays were from Clontech. The coding sequence of *FT* was cloned into pGBKT7 to produce BD-FT, which was used as a bait to screen cDNA library (CD4-30 from ABRC) for identifying interacting proteins of FT. Selection was performed on medium lacking histidine, tryptophan, and leucine (SD-His/-Trp/-Leu) supplemented with 30 mM 3-amino-1, 2, 4-triazole. To verify the interaction between FT and FTIP1, various versions of *FTIP1* coding sequences were cloned into pGADT7. The resulting vectors were co-transformed with BD-FT, and the transformed cells were selected on SD-His/-Trp/-Leu medium supplemented with 30 mM 3-amino-1, 2, 4-triazole. β-galactosidase assays were performed according to the Yeast Protocols Handbook (Clontech).

### GST In Vitro Pull-Down Assay

The cDNA encoding FT was cloned into the pGEX-4T-1 vector (Pharmacia) and introduced into *E. coli* Rosetta (DE3) (Novagen). Transformed cells were cultured until the OD_600 nm_ reached 0.6, and IPTG was added to a final concentration of 0.6 mM to start induction. After overnight induction at 16°C, cells were collected and homogenized with lysis buffer (10 mM Tris-HCl pH 8.0, 150 mM NaCl, 1 mM EDTA, 1% Triton-100, and 10 mM PMSF). The soluble GST fusion proteins were extracted and immobilized on glutathione sepharose beads (Amersham Biosciences) for subsequent pull-down assays. The FTIP1 N-terminal fragment containing the three C2 domains (N501) was cloned into pGBKT7 vector (Clontech). The resulting plasmid was added to the TNT T7 Quick Coupled Transcription/Translation Systems (Promega) to synthesize myc-FTIP1(N501) protein. The resulting fusion protein was then incubated with the immobilized GST and GST fusion proteins. Proteins retained on the beads were resolved by SDS-PAGE and detected with anti-myc antibody (Santa Cruz Biotechnology).

### Transient Expression of Proteins in *Nicotiana Benthamiana* Leaf Epidermal Cells

The overnight *Agrobacterium* cultures with a desired expression vector (*35S:FTIP1:GFP*, various RFP- or CFP-tagged organelle markers, *35S:FT:RFP*, or *35S:GFP*) were harvested and resuspended with infiltration buffer (10 mM MES pH 5.6, 10 mM MgCl_2_, and 100 µM acetosyringone) with OD_600 nm_ at 0.4. To compare protein localization, equal volumes of infiltration solutions containing different expression vectors were mixed together and incubated for 3 h at room temperature. Infiltration solutions were infiltrated into the abaxial surface of 3-wk-old tobacco (*Nicotiana benthamiana*) leaves with syringes. The leaves were examined 2 d after infiltration under a confocal microscope.

### GUS Staining

Tissues were infiltrated with staining solution (50 mM sodium phosphate buffer, pH 7.0, 0.5 mM potassium ferrocyanide, 0.5 mM potassium ferricyanide, and 0.5 mg/mL X-Gluc) in a vacuum chamber, and subsequently incubated with staining solution at 37°C overnight. For sectioning, samples were dehydrated through an ethanol series, an ethanol/histoclear series, and finally embedded in paraplast (Sigma). Samples were then orientated and sectioned at a thickness of 3 µm with a microtome.

### Expression Analysis

Total RNA was isolated with RNeasy Plant Mini Kit (Qiagen) and reverse-transcribed with ThermoScript RT-PCR System (Invitrogen) according to the manufacturers' instructions. Real-time PCR was performed in triplicates on 7900HT Fast Real-Time PCR system (Applied Biosystems) with SYBR Green PCR Master Mix (Applied Biosystems). The difference between the cycle threshold (Ct) of the target gene and the Ct of *TUB2* (ΔCt = Ct_target gene_−Ct_tubulin_) was used to obtain the normalized expression of target genes, which corresponded to 2^−ΔCt^. Expression analysis was performed with at least three biological replicates. Primers for real-time PCR are listed in Table S2.

### In Vivo Protein Interaction Assay

Plant tissues were collected and fixed with ice-cold 4% paraformaldehyde (PFA; Sigma-Aldrich) at pH 7.0 in a vacuum chamber. A serial PFA/sucrose change was applied till the tissues were finally equilibrated in PFA with 20% sucrose. Tissues were then embedded in 1.5% agarose gel, placed onto the microtome tissue holder, and flash frozen with liquid nitrogen. Tissues were cut in cryo-microtome with 20 µm thickness, and the sections were placed on slides. After complete drying, the slides were rehydrated with 100 mM Tris pH 8, 50 mM EDTA, and permeabilized with proteinase K (1 µg/ml) in the same buffer for 10 min at room temperature. Slides were washed with 2 mg/ml glycine followed by washing with PBS solution. Chlorophyll molecules were subsequently removed by incubating the slides with 1∶1 acetone/methanol mixture twice for 5 min. After drying, slides were rehydrated with PBS and finally treated with 4% paraformaldehyde for 10 min followed by washing with PBS solution.

In situ Proximity Ligation Assay (PLA) was performed with Duolink kit (Olink Bioscience) with minor modifications. The above treated slides were firstly blocked with 2% Bovine Serum Albumin in 100 mM Tris pH 7.5, 150 mM NaCl, and 0.3% Triton X-100 for 45 min at 37°C, and probed with the mixture of anti-GFP and anti-HA antibodies diluted in the blocking solution (1∶60) for 45 min at 37°C. The slides were washed three times and probed with diluted PLUS and MINUS PLA probes for 1 h at 37°C and subsequently washed 5 times. The slides were further incubated with the ligation solution, washed, and subsequently incubated with the amplification-polymerase solution with all components provided in the kit. After signal amplification, the slides were washed and mounted with PBS solution for further observation.

### Immunogold Transmission Electron Microscopy

Samples were fixed with paraformaldehyde-glutaraldehyde solution (2% and 2.5%, respectively) and imbedded with LR white medium (EMS). Ultra-thin sections (85 nm) were cut and mounted on nickel grids. The grids were blocked with 1% BSA in TTBS (20 mM Tris, 500 mM NaCl, and 0.05% Tween-20, pH 7.5) for 30 min and subsequently incubated with anti-HA or anti-myc antibody at 1∶5 (v/v) for 1 h at room temperature. The grids were washed with TTBS for three times and further incubated with 15 nm gold-conjugated goat anti-mouse antibody (EMS) that was diluted 1∶20 with blocking solution. After 40 min of incubation, the grids were washed with TTBS for three times and with distilled water twice. Tissue staining was performed with 2% uranyl acetate for 15 min at room temperature, and pictures were taken by transmission electron microscope (Jeol JEM-1230).

For quantitative analysis of immunogold labeling, micrographs of randomly photographed immunogold-labeled transverse sections of the first rosette leaves of 15-d-old seedlings with various genetic backgrounds were measured as previously reported [32]. The data were presented as the mean number of gold particles per µm^2^ plus or minus standard deviation. The projected cell area was measured by a LI-3100C area meter (Li-Cor). We analyzed 56 individual sections from eight different leaves of each genotype for calculating the density of gold particles over the projected cell area. Statistical analysis was performed using a two-tailed unpaired Student's *t* test. Two-tailed test results were considered statistically significant at *p*<0.05.

## Supporting Information

Figure S1 Bioinformatic analysis of FTIP1 protein sequence. (A) Schematic drawing of the FTIP1 protein. Three C2 domains and the PRT_C domain are shown as red and blue boxes, respectively. The bar above the scheme indicates the fragment isolated from the yeast two-hybrid screening. (B) Topology prediction of the transmembrane region in FTIP1 using the TopPred program (http://mobyle.pasteur.fr/cgi-bin/portal.py?form=toppred).
(TIF)

Figure S2 *FTIP1* is specifically expressed in vascular tissues. (A) Schematic diagrams of *gFTIP1* and *FTIP1:GUS* constructs. A 5.1 kb *FTIP1* genomic fragment (*gFTIP1*) including the 2.1 kb upstream sequence, 2.4 kb coding sequence (CDS), and 0.6 kb downstream sequence was able to rescue the late-flowering phenotype of *ftip1-1* as shown in Figure 1E. To examine the detailed expression pattern of *FTIP1*, we generated the construct *FTIP1:GUS*, in which the same 2.1 kb *FTIP1* upstream sequence included in *gFTIP1* for the gene complementation test was fused to the *GUS* reporter gene. (B–H) GUS staining of various tissues of *FTIP1:GUS*. (B) A 3-d-old seedling. (C) The shoot apex of a 3-d-old seedling. Asterisk indicates the shoot apical meristem. (D) An inflorescence apex. (E) A cauline leaf with an auxiliary shoot. (F) A cross-section of an inflorescence stem. (G) An open flower. (H) A silique. Bars: (C), 20 µm; (F), 200 µm.
(TIF)

Figure S3 Quantitative real-time PCR analysis of *FTIP1* expression in various tissues of wild-type plants. Plant tissues were collected from 40-d-old wild-type plants. Results were normalized against the expression of *TUB2* based on three biological replicates. Error bars indicate SD.
(TIF)

Figure S4 *FTIP1* mRNA expression is not regulated by photoperiod, GA, and vernalization pathways. (A) *FTIP1* expression is not significantly changed in wild-type plants grown under long days (LDs) and short days (SDs). (B) *FTIP1* expression is not affected by vernalization treatment. For vernalization treatment, seeds were grown on MS medium and vernalized at 4°C under low light condition for 8 wk. 9-d-old seedlings grown under LDs were harvested for expression analysis. (C) *FTIP1* expression is not affected by gibberellin (GA) treatment. Wild-type plants grown under SDs were treated weekly with 100 µM GA. Seedlings treated for 1 wk (1 w) or 3 wk (3 w) were harvested for expression analysis. (D) *ftip1-1* and wild-type plants exhibit similar flowering time in response to GA treatment. *ftip1-1* and wild-type plants grown under SDs were treated weekly with 100 µM GA. (E) *FTIP1* expression levels do not obviously oscillate within a 24-h cycle under LDs. 9-d-old wild-type plants grown under LDs were harvested at 2-h intervals over a 24-h period. Sampling time was expressed in hours as Zeitgeber time (ZT), which is the number of hours after the onset of illumination. The lowest expression level of each gene is set as 1. Gene expression in (A–C) and (E) was determined by quantitative real-time PCR and normalized against *TUB2* levels. Error bars indicate SD.
(TIF)

Figure S5 *FTIP1* mRNA expression is not obviously altered in various flowering time mutants. (A) *FTIP1* expression in photoperiod-pathway mutants. (B) *FTIP1* expression in autonomous-pathway mutants. (C) *FTIP1* expression in several other flowering time mutants. 9-d-old wild-type and mutant seedlings grown under LDs were harvested for expression analysis by quantitative real-time PCR. Results were normalized against the expression of *TUB2*. Error bars indicate SD.
(TIF)

Figure S6 Subcellular colocalization of FTIP1:GFP and the ER marker in *N. benthamiana* leaf epidermal cells. Bar, 20 µm.
(TIF)

Figure S7 Control experiments for measuring 4HA:FTIP1 localization by immunogold electron microscopy. (A) Western blot analysis showing that the 4HA:FTIP1 protein is intact. As the crude extract did not generate any signal, the sample was enriched with anti-HA agarose conjugate and used for SDS-PAGE analysis. The membrane was probed with anti-HA antibody. Lane 1, wild-type seedlings; Lane 2, *FTIP1:4HA:FTIP1 ftip1-1* seedlings. (B) Quantitative analysis of immunogold signals revealed by immunogold electron microscopy of *FTIP1:4HA:FTIP1 ftip1-1* transgenic plants shows that anti-HA antibody could specifically recognize 4HA:FTIP1. The left panel shows the quantification of 4HA:FTIP1 immunogold signals or immunogold background signals in CC and PD of *FTIP1:4HA:FTIP1 ftip1-1* probed with anti-HA antibody or mouse IgG control. The data are presented as the mean number of gold particles per µm^2^ with standard deviation. Statistical analysis was performed using a two-tailed unpaired Student's *t* test. The results are considered statistically significant at *p*<0.05. The middle and right panels show the frequency histograms of appearance of 4HA:FTIP1 immunogold signals and immunogold background signals in *FTIP1:4HA:FTIP1 ftip1-1* probed with anti-HA antibody and mouse IgG, respectively. Asterisks indicate that in all sections examined using IgG control, the number and frequency of PD with gold particles are zero. (C) Immunogold electron microscopy of CC-SE complexes in wild-type plants using anti-HA antibody. Left panel, a representative CC-SE complex. Bar, 1 µm. Middle panel, density of immunogold background signals observed in CC and PD of wild-type plants probed with anti-HA antibody. Right panel, frequency histogram of appearance of immunogold background signals in CC and PD of wild-type plants probed with anti-HA antibody in all examined sections. Asterisks indicate that in all sections examined using anti-HA antibody, the number and frequency of PD with gold particles are zero. CC, companion cell; PD, plasmodesmata; SE, sieve element.
(TIF)

Figure S8 Colocalization of FTIP1:GFP and FT:RFP in *N. benthamiana* leaf epidermal cells. GFP, GFP fluorescence; RFP, RFP fluorescence; Merge, merge of GFP and RFP; Nu, nucleus. Bar, 10 µm.
(TIF)

Figure S9 Change in FTIP1 activity does not affect free GFP distribution. (A) Confocal analysis of free GFP protein distribution in the apical region of 11-d-old *SUC2:GFP* seedlings in different genetic backgrounds. Bar, 20 µm. (B) Confocal analysis of free GFP protein distribution in the primary vein of the first rosette leaves from 11-d-old *SUC2: GFP* seedlings in different genetic backgrounds. Bar, 20 µm. GFP, GFP fluorescence; BF, bright field image; Merge, merge of GFP and BF.
(TIF)

Figure S10 Control experiments for measuring FT:9myc localization by immunogold electron microscopy. (A) Analysis of FT:9myc distribution in CC-SE complexes of the phloem in the first rosette leaves of 15-d-old *SUC2:FT:9myc* and *SUC2:FT:9myc ftip1-1* seedlings by immunogold electron microscopy using mouse IgG antibody. All tissues examined show similar background signals generated by IgG antibody. Left panel, representative CC-SE complexes from *SUC2:FT:9myc* and *SUC2:FT:9myc ftip1-1* including higher magnification views of CCs and SEs. Bars: 2 µm in the left panels; 0.5 µm in the magnified views. Middle panel, density of immunogold background signals observed in CCs and SEs of *SUC2:FT:9myc* (WT background) and *SUC2:FT:9myc ftip1-1* (*ftip1-1* background). The data are presented as the mean number of immunogold background particles per µm^2^ with standard deviation. Right panel, frequency histogram of appearance of immunogold background signals in CCs and SEs in all examined sections. CC, companion cell; SE, sieve element. (B) Analysis of FT:9myc distribution in xylem parenchyma cells of the first rosette leaves of 15-d-old *SUC2:FT:9myc* and *SUC2:FT:9myc ftip1-1* seedlings by immunogold electron microscopy using anti-myc antibody. The results show that anti-myc antibody does not generate non-specific signal in xylem parenchyma cells. Left panel, representative xylem parenchyma cells from *SUC2:FT:9myc* (WT background) and *SUC2:FT:9myc ftip1-1* (*ftip1-1* background). Bar, 2 µm. Middle panel, density of gold particles observed in xylem parenchyma cells. The data are presented as the mean number of gold particles per µm^2^ with standard deviation. Right panel, frequency histogram of appearance of immunogold signals in xylem parenchyma cells in all examined sections. XP, xylem parenchyma; XV, xylem vessel. (C) Analysis of immunogold background signals in CC-SE complexes of the phloem in the first rosette leaves of 15-d-old wild-type seedlings by immunogold electron microscopy using anti-myc antibody. All tissues examined show similar background signals. Left panel, a representative CC-SE complex including higher magnification views of CC and SE. Bars, 1 µm. Middle panel, density of immunogold background signals observed in CCs and SEs of wild-type plants probed with anti-myc antibody. Right panel, frequency histogram of appearance of immunogold background signals in CCs and SEs in all examined sections. CC, companion cell; SE, sieve element.
(TIF)

Figure S11 Overexpression of *FTIP1* causes late flowering. (A) Distribution of flowering time in *35S:FTIP1* T1 transgenic plants. Among 28 independent lines generated, all of them show different degrees of late flowering. (B) Homozygous transgenic plants (T3 generation) of three representative *35S:FTIP1* lines consistently show late flowering. *35S:FTIP1 #1*, *35S:FTIP1 #2*, and *35S:FTIP1 #3* exhibit weak, moderate, and strong flowering phenotypes, respectively. Error bars indicate SD. (C) *FTIP1* expression is elevated in *35S:FTIP1* lines. The degrees of late flowering in *35S:FTIP1* shown in (B) are not related to the elevated levels of *FTIP1* in *35S:FTIP1 #1*, *35S:FTIP1 #2*, and *35S:FTIP1 #3*. 9-d-old wild-type and transgenic seedlings grown under LDs were harvested for expression analysis by quantitative real-time PCR. Results were normalized against the expression of *TUB2*. Error bars indicate SD.
(TIF)

Figure S12 Expression of several key flowering genes in *ftip1-1*. (A) Expression of *AP1*, *SOC1*, and *FT* is downregulated in *ftip1-1*. 9-d-old wild-type and *ftip1-1* seedlings grown under LDs were harvested for expression analysis. The gene expression in wild-type plants is set as 1. (B) *CO* expression is not significantly changed in *ftip1-1* within a 24-h cycle under LDs. (C) *FT* expression is consistently downregulated in *ftip1-1* within a 24-h cycle under LDs. In (B and C), 9-d-old wild-type and *ftip1-1* seedlings grown under LDs were harvested at 2-h intervals over a 24-h period for expression analysis. Gene expression in (A–C) was determined by quantitative real-time PCR and normalized against *TUB2* levels. Error bars indicate SD.
(TIF)

Figure S13 Phylogenetic analysis of FTIP1-like proteins. (A) Phylogenetic tree showing FTIP1 homologs and synaptotagmins in *Arabidopsis*. The phylogenetic tree was generated based on the protein alignment of FTIP1, its 16 *Arabidopsis* homologs, and three *Arabidopsis* synaptotagmins (SYTA, SYTB, and SYTC). The scale bar represents 0.1 amino acid substitution. (B) Phylogenetic analysis of FTIP1-like proteins in different plant species. The phylogenetic tree was constructed with the neighbor-joining algorithm using the program MEGA 5.05 based on the alignment of the amino acid sequences of FTIP1-like proteins. Each terminal node of the tree is labeled by the two-letter abbreviation of the corresponding species name and the unique identifier. Bootstrap values (>50%) in 500 replicates are indicated next to the nodes. Zm, *Zea mays*; Os, *Oryza sativa*; Mt, *Medicago truncatula*; Pt, *Populus trichocarpa*; Bd, *Brachypodium distachyon*.
(TIF)

Table S1 List of potential FT-interacting proteins isolated from the yeast two-hybrid screening.
(PDF)

Table S2 Primers used for expression analyses in this study.
(PDF)

## Abbreviations

AP1: APETALA1
ER: endoplasmic reticulum
FT: FLOWERING LOCUS T
FTIP1: FT-INTERACTING PROTEIN 1
GFP: green fluorescent protein
GUS: β-glucuronidase
LDs: long days
PLA: proximity ligation assay
PRT_C: phosphoribosyltransferase C-terminal
SAM: shoot apical meristem
SDs: short days
SOC1: SUPPRESSOR OF OVEREXPRESSION OF CONSTANS 1
SUC2: SUCROSE TRANSPORTER 2
TSF: TWIN SISTER OF FT
