# Supplementary figures and images for: FTIP1 Is an Essential Regulator Required for Florigen Transport
Source: PLoS Biol. 2012 Apr 17;10(4):e1001313. doi: 10.1371/journal.pbio.1001313 (PMC3328448; doi:10.1371/journal.pbio.1001313)

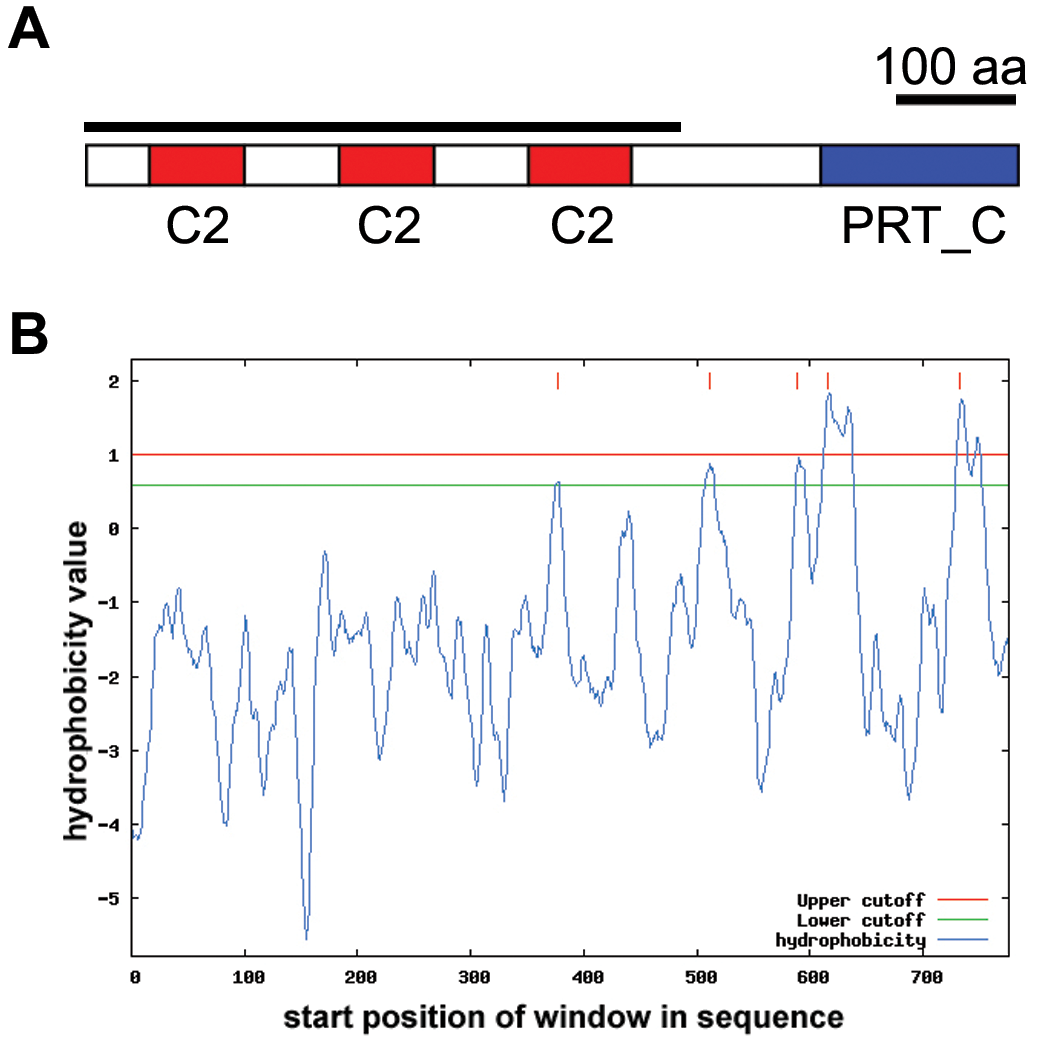

Supplement: Figure S1 — Bioinformatic analysis of FTIP1 protein sequence. (A) Schematic drawing of the FTIP1 protein. Three C2 domains and the PRT_C domain are shown as red and blue boxes, respectively. The bar above the scheme indicates the fragment isolated from the yeast two-hybrid screening. (B) Topology prediction of the transmembrane region in FTIP1 using the TopPred program (http://mobyle.pasteur.fr/cgi-bin/portal.py?form=toppred). (TIF) [file pbio.1001313.s001.tif]

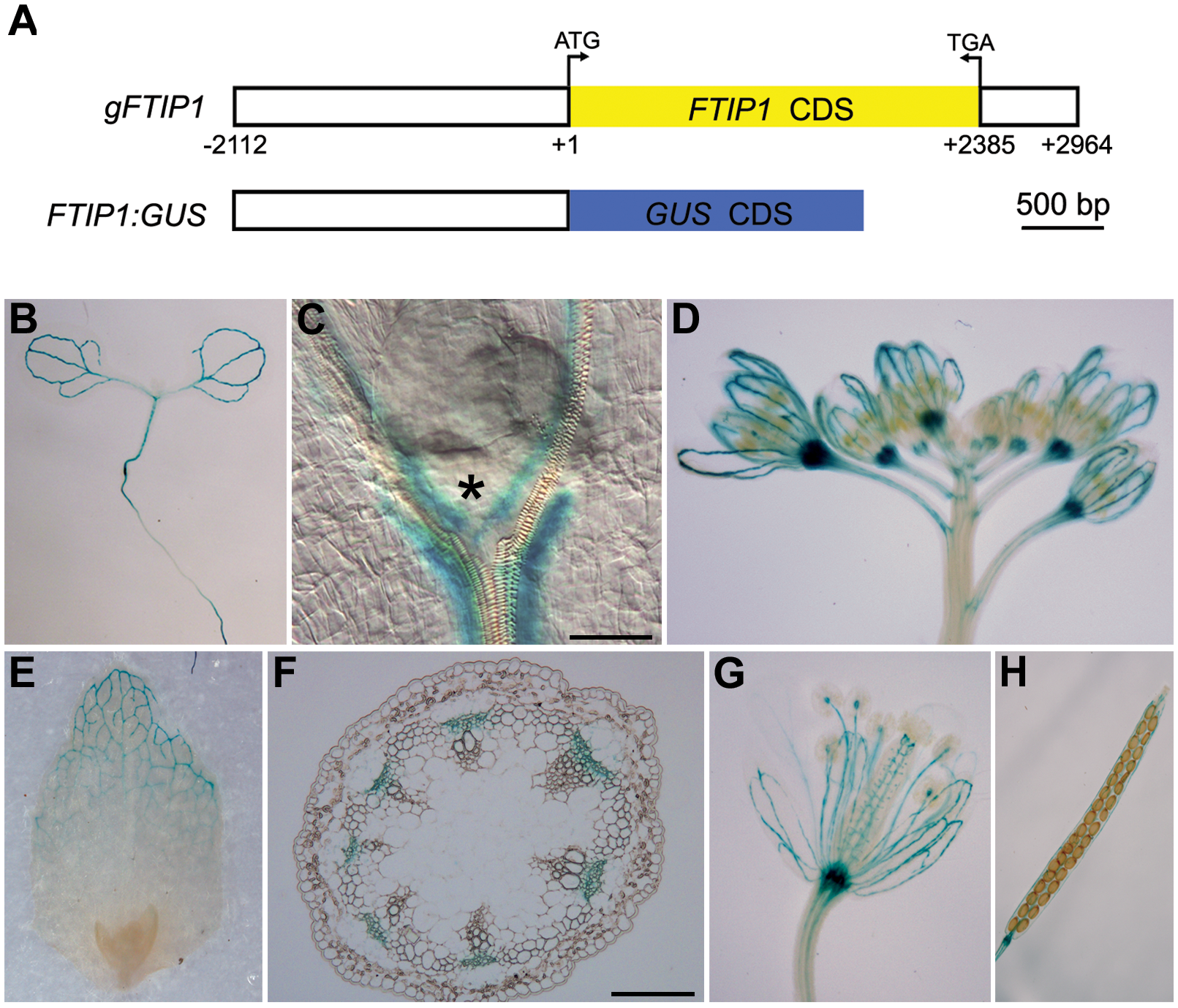

Supplement: Figure S2 — FTIP1 is specifically expressed in vascular tissues. (A) Schematic diagrams of gFTIP1 and FTIP1:GUS constructs. A 5.1 kb FTIP1 genomic fragment (gFTIP1) including the 2.1 kb upstream sequence, 2.4 kb coding sequence (CDS), and 0.6 kb downstream sequence was able to rescue the late-flowering phenotype of ftip1-1 as shown in Figure 1E. To examine the detailed expression pattern of FTIP1, we generated the construct FTIP1:GUS, in which the same 2.1 kb FTIP1 upstream sequence included in gFTIP1 for the gene complementation test was fused to the GUS reporter gene. (B–H) GUS staining of various tissues of FTIP1:GUS. (B) A 3-d-old seedling. (C) The shoot apex of a 3-d-old seedling. Asterisk indicates the shoot apical meristem. (D) An inflorescence apex. (E) A cauline leaf with an auxiliary shoot. (F) A cross-section of an inflorescence stem. (G) An open flower. (H) A silique. Bars: (C), 20 µm; (F), 200 µm. (TIF) [file pbio.1001313.s002.tif]

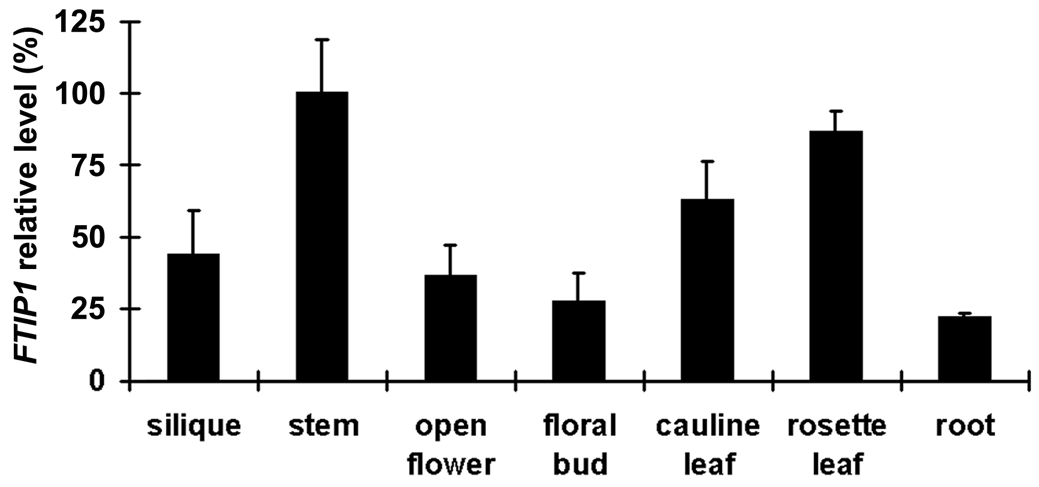

Supplement: Figure S3 — Quantitative real-time PCR analysis of FTIP1 expression in various tissues of wild-type plants. Plant tissues were collected from 40-d-old wild-type plants. Results were normalized against the expression of TUB2 based on three biological replicates. Error bars indicate SD. (TIF) [file pbio.1001313.s003.tif]

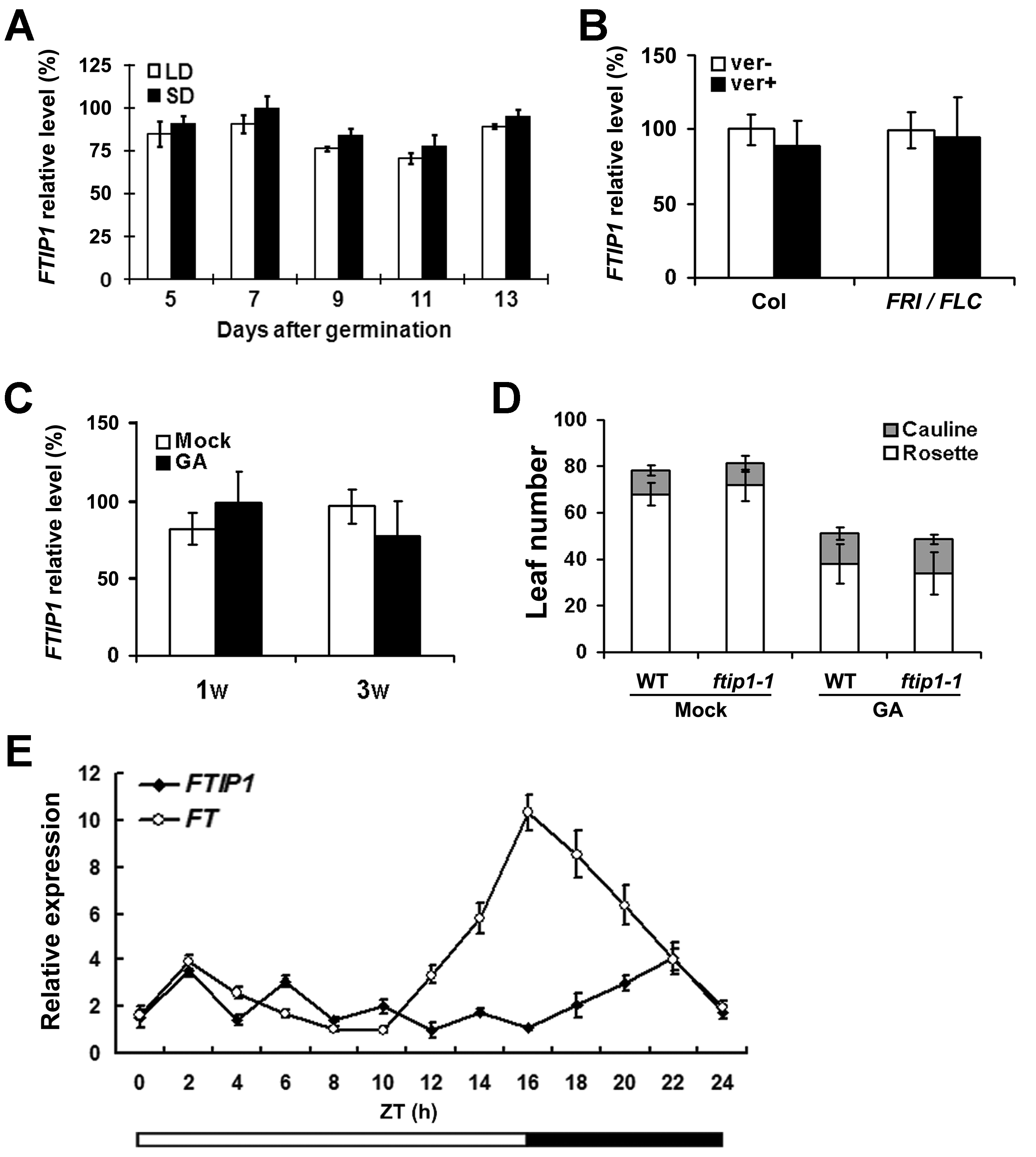

Supplement: Figure S4 — FTIP1 mRNA expression is not regulated by photoperiod, GA, and vernalization pathways. (A) FTIP1 expression is not significantly changed in wild-type plants grown under long days (LDs) and short days (SDs). (B) FTIP1 expression is not affected by vernalization treatment. For vernalization treatment, seeds were grown on MS medium and vernalized at 4°C under low light condition for 8 wk. 9-d-old seedlings grown under LDs were harvested for expression analysis. (C) FTIP1 expression is not affected by gibberellin (GA) treatment. Wild-type plants grown under SDs were treated weekly with 100 µM GA. Seedlings treated for 1 wk (1 w) or 3 wk (3 w) were harvested for expression analysis. (D) ftip1-1 and wild-type plants exhibit similar flowering time in response to GA treatment. ftip1-1 and wild-type plants grown under SDs were treated weekly with 100 µM GA. (E) FTIP1 expression levels do not obviously oscillate within a 24-h cycle under LDs. 9-d-old wild-type plants grown under LDs were harvested at 2-h intervals over a 24-h period. Sampling time was expressed in hours as Zeitgeber time (ZT), which is the number of hours after the onset of illumination. The lowest expression level of each gene is set as 1. Gene expression in (A–C) and (E) was determined by quantitative real-time PCR and normalized against TUB2 levels. Error bars indicate SD. (TIF) [file pbio.1001313.s004.tif]

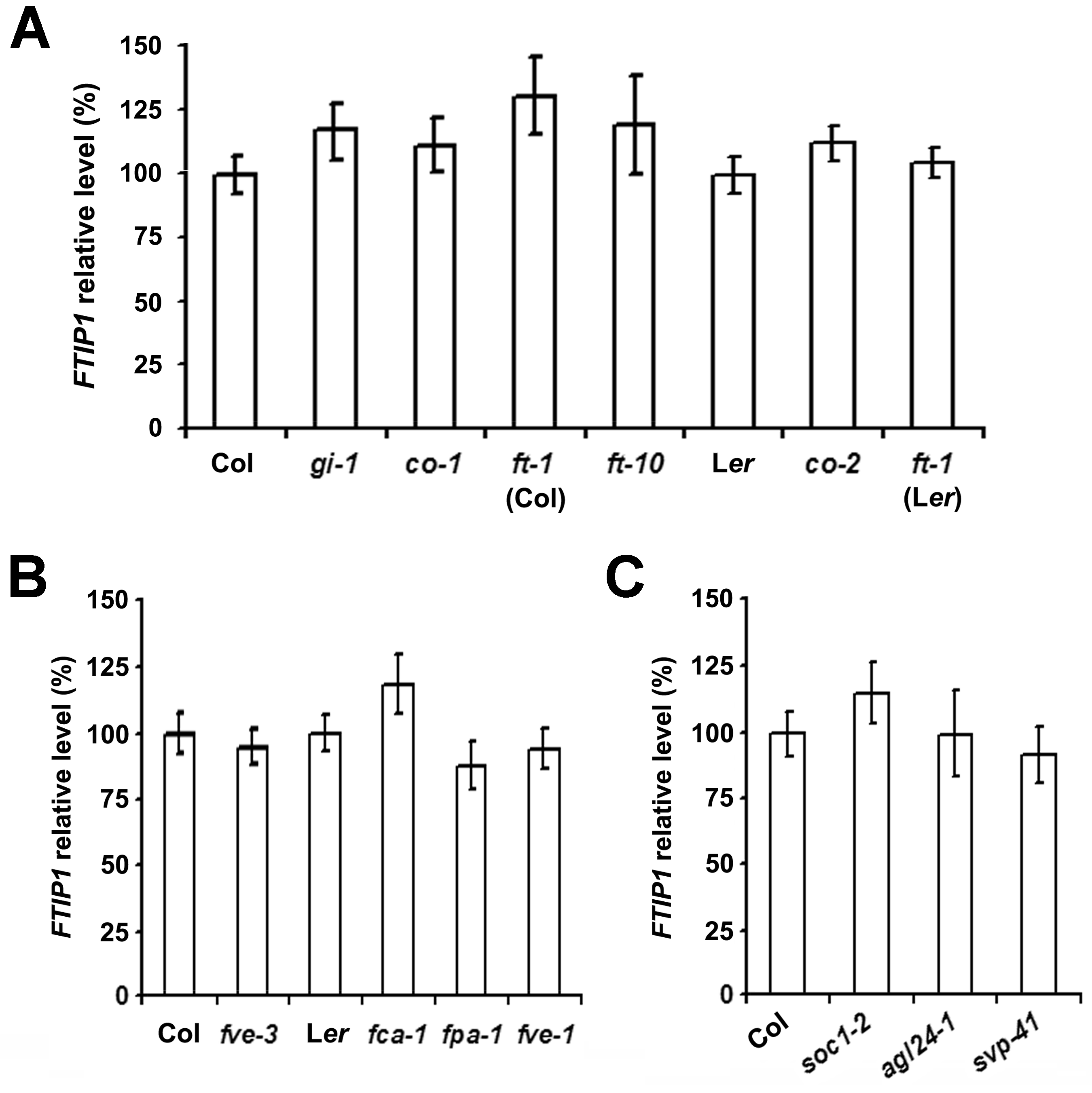

Supplement: Figure S5 — FTIP1 mRNA expression is not obviously altered in various flowering time mutants. (A) FTIP1 expression in photoperiod-pathway mutants. (B) FTIP1 expression in autonomous-pathway mutants. (C) FTIP1 expression in several other flowering time mutants. 9-d-old wild-type and mutant seedlings grown under LDs were harvested for expression analysis by quantitative real-time PCR. Results were normalized against the expression of TUB2. Error bars indicate SD. (TIF) [file pbio.1001313.s005.tif]

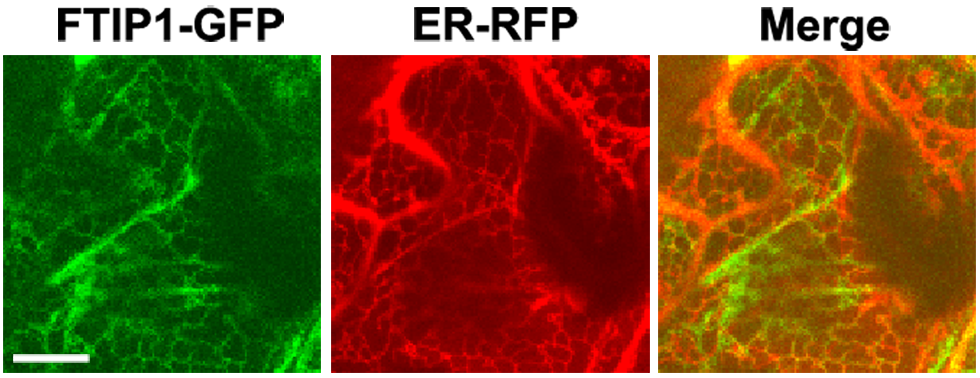

Supplement: Figure S6 — Subcellular colocalization of FTIP1:GFP and the ER marker in N. benthamiana leaf epidermal cells. Bar, 20 µm. (TIF) [file pbio.1001313.s006.tif]

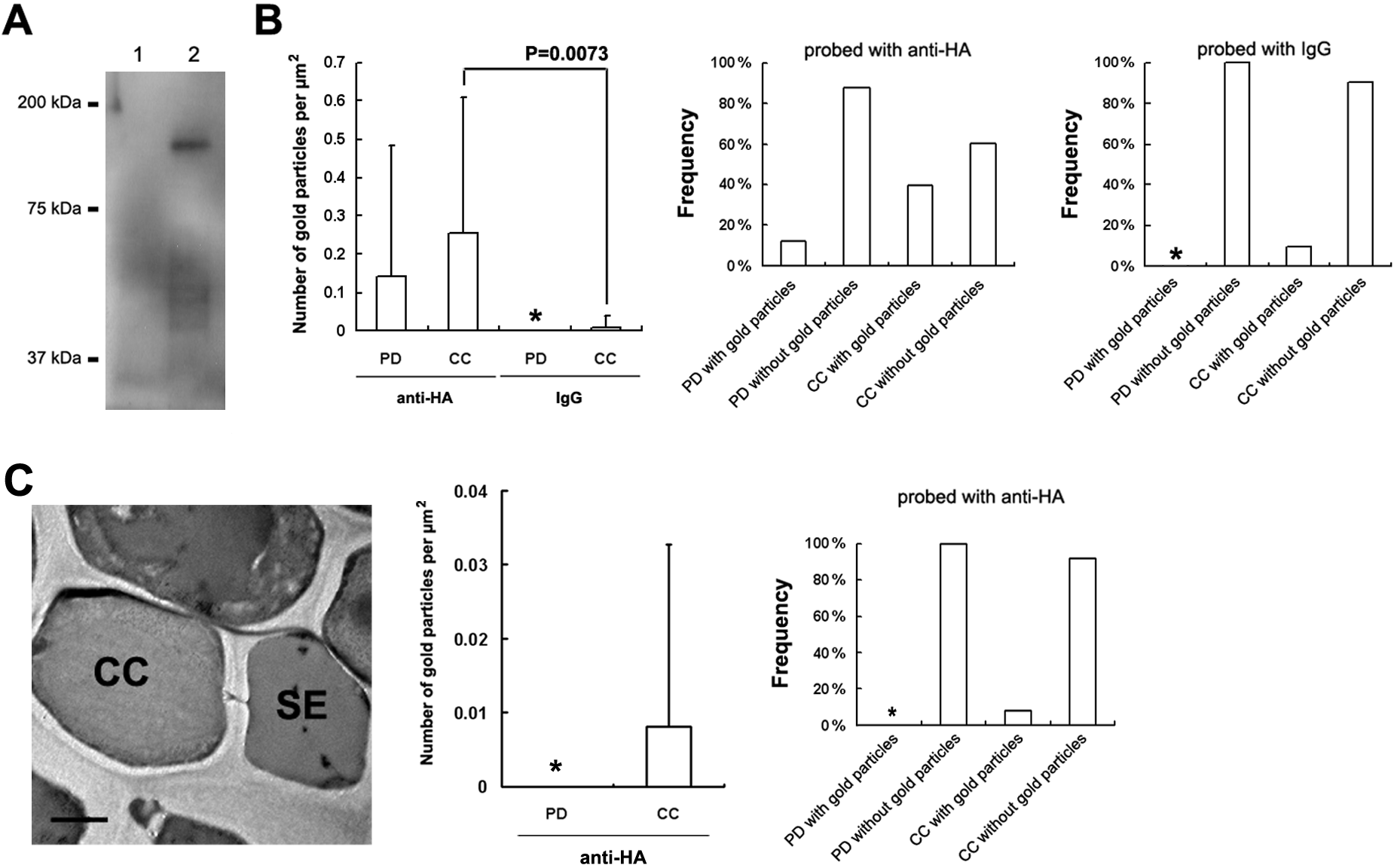

Supplement: Figure S7 — Control experiments for measuring 4HA:FTIP1 localization by immunogold electron microscopy. (A) Western blot analysis showing that the 4HA:FTIP1 protein is intact. As the crude extract did not generate any signal, the sample was enriched with anti-HA agarose conjugate and used for SDS-PAGE analysis. The membrane was probed with anti-HA antibody. Lane 1, wild-type seedlings; Lane 2, FTIP1:4HA:FTIP1 ftip1-1 seedlings. (B) Quantitative analysis of immunogold signals revealed by immunogold electron microscopy of FTIP1:4HA:FTIP1 ftip1-1 transgenic plants shows that anti-HA antibody could specifically recognize 4HA:FTIP1. The left panel shows the quantification of 4HA:FTIP1 immunogold signals or immunogold background signals in CC and PD of FTIP1:4HA:FTIP1 ftip1-1 probed with anti-HA antibody or mouse IgG control. The data are presented as the mean number of gold particles per µm2 with standard deviation. Statistical analysis was performed using a two-tailed unpaired Student's t test. The results are considered statistically significant at p<0.05. The middle and right panels show the frequency histograms of appearance of 4HA:FTIP1 immunogold signals and immunogold background signals in FTIP1:4HA:FTIP1 ftip1-1 probed with anti-HA antibody and mouse IgG, respectively. Asterisks indicate that in all sections examined using IgG control, the number and frequency of PD with gold particles are zero. (C) Immunogold electron microscopy of CC-SE complexes in wild-type plants using anti-HA antibody. Left panel, a representative CC-SE complex. Bar, 1 µm. Middle panel, density of immunogold background signals observed in CC and PD of wild-type plants probed with anti-HA antibody. Right panel, frequency histogram of appearance of immunogold background signals in CC and PD of wild-type plants probed with anti-HA antibody in all examined sections. Asterisks indicate that in all sections examined using anti-HA antibody, the number and frequency of PD with gold particles are zero. CC, comp [file pbio.1001313.s007.tif]

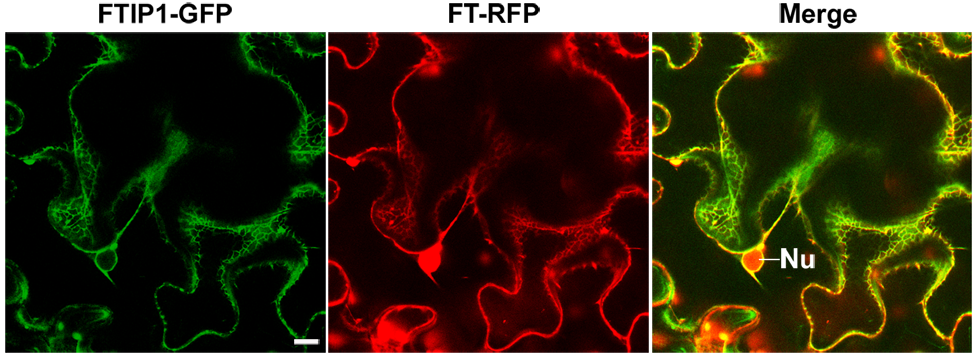

Supplement: Figure S8 — Colocalization of FTIP1:GFP and FT:RFP in N. benthamiana leaf epidermal cells. GFP, GFP fluorescence; RFP, RFP fluorescence; Merge, merge of GFP and RFP; Nu, nucleus. Bar, 10 µm. (TIF) [file pbio.1001313.s008.tif]

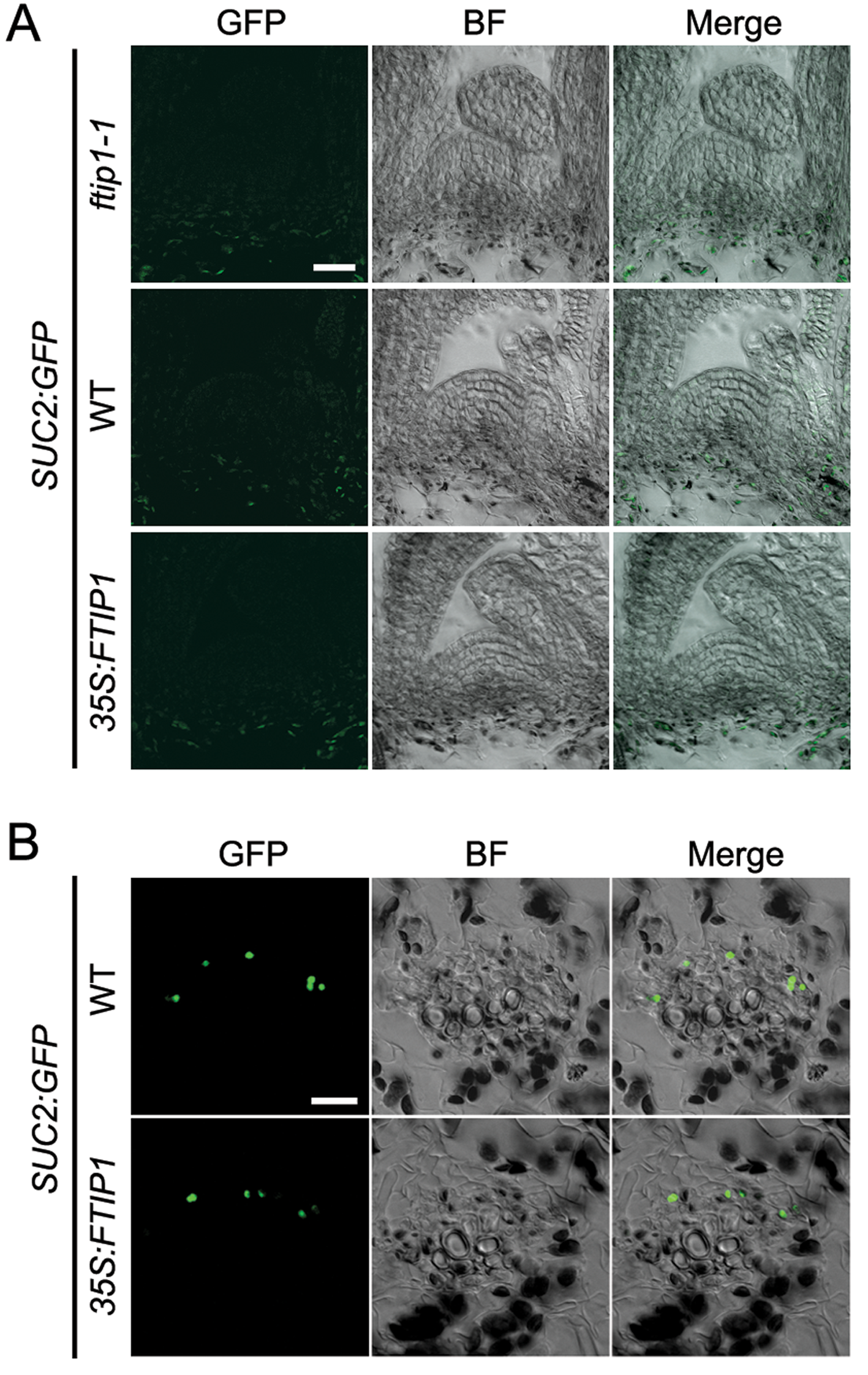

Supplement: Figure S9 — Change in FTIP1 activity does not affect free GFP distribution. (A) Confocal analysis of free GFP protein distribution in the apical region of 11-d-old SUC2:GFP seedlings in different genetic backgrounds. Bar, 20 µm. (B) Confocal analysis of free GFP protein distribution in the primary vein of the first rosette leaves from 11-d-old SUC2: GFP seedlings in different genetic backgrounds. Bar, 20 µm. GFP, GFP fluorescence; BF, bright field image; Merge, merge of GFP and BF. (TIF) [file pbio.1001313.s009.tif]

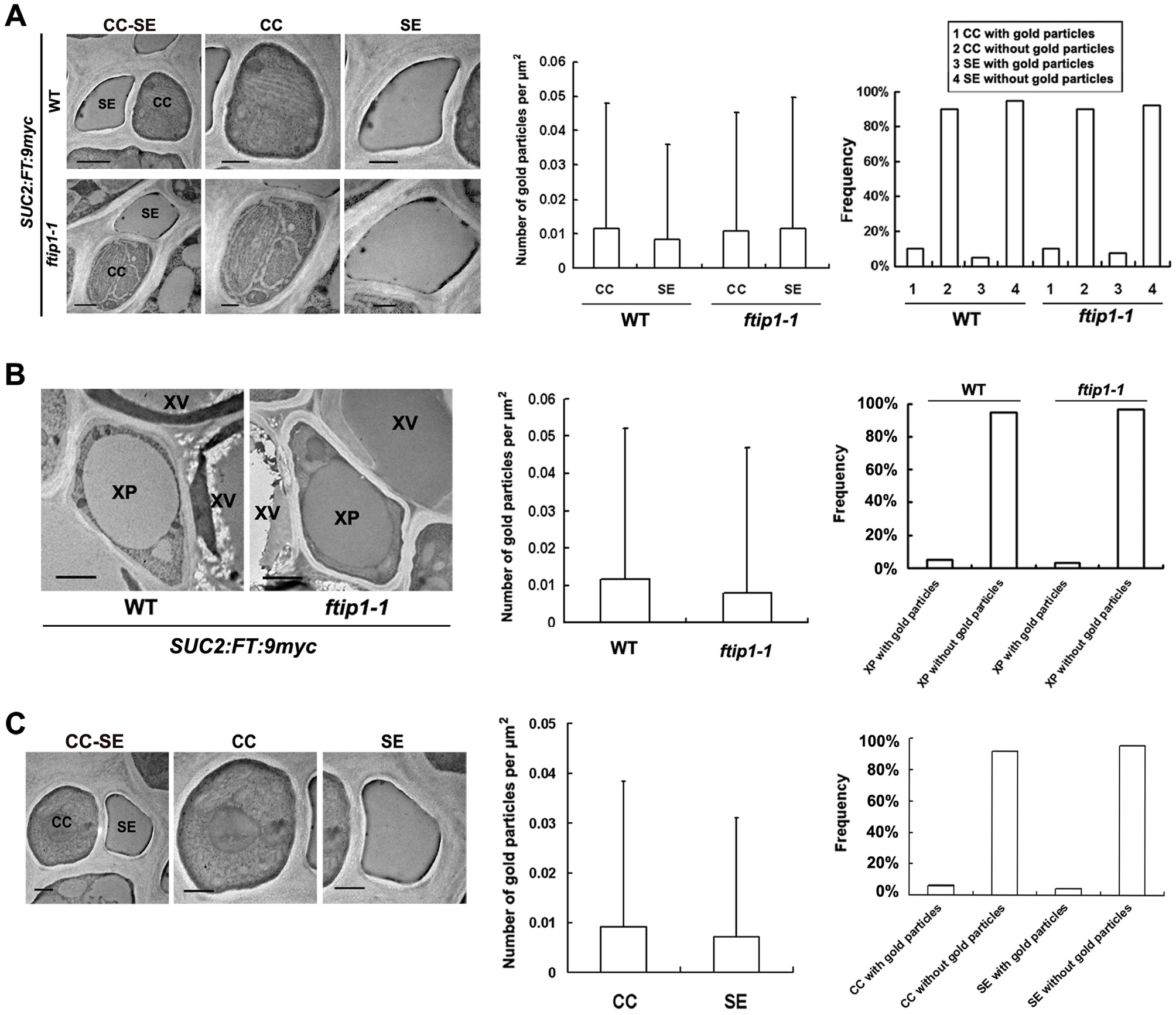

Supplement: Figure S10 — Control experiments for measuring FT:9myc localization by immunogold electron microscopy. (A) Analysis of FT:9myc distribution in CC-SE complexes of the phloem in the first rosette leaves of 15-d-old SUC2:FT:9myc and SUC2:FT:9myc ftip1-1 seedlings by immunogold electron microscopy using mouse IgG antibody. All tissues examined show similar background signals generated by IgG antibody. Left panel, representative CC-SE complexes from SUC2:FT:9myc and SUC2:FT:9myc ftip1-1 including higher magnification views of CCs and SEs. Bars: 2 µm in the left panels; 0.5 µm in the magnified views. Middle panel, density of immunogold background signals observed in CCs and SEs of SUC2:FT:9myc (WT background) and SUC2:FT:9myc ftip1-1 (ftip1-1 background). The data are presented as the mean number of immunogold background particles per µm2 with standard deviation. Right panel, frequency histogram of appearance of immunogold background signals in CCs and SEs in all examined sections. CC, companion cell; SE, sieve element. (B) Analysis of FT:9myc distribution in xylem parenchyma cells of the first rosette leaves of 15-d-old SUC2:FT:9myc and SUC2:FT:9myc ftip1-1 seedlings by immunogold electron microscopy using anti-myc antibody. The results show that anti-myc antibody does not generate non-specific signal in xylem parenchyma cells. Left panel, representative xylem parenchyma cells from SUC2:FT:9myc (WT background) and SUC2:FT:9myc ftip1-1 (ftip1-1 background). Bar, 2 µm. Middle panel, density of gold particles observed in xylem parenchyma cells. The data are presented as the mean number of gold particles per µm2 with standard deviation. Right panel, frequency histogram of appearance of immunogold signals in xylem parenchyma cells in all examined sections. XP, xylem parenchyma; XV, xylem vessel. (C) Analysis of immunogold background signals in CC-SE complexes of the phloem in the first rosette leaves of 15-d-old wild-type seedlings by immunogold electron microscopy using anti-myc antibody [file pbio.1001313.s010.tif]

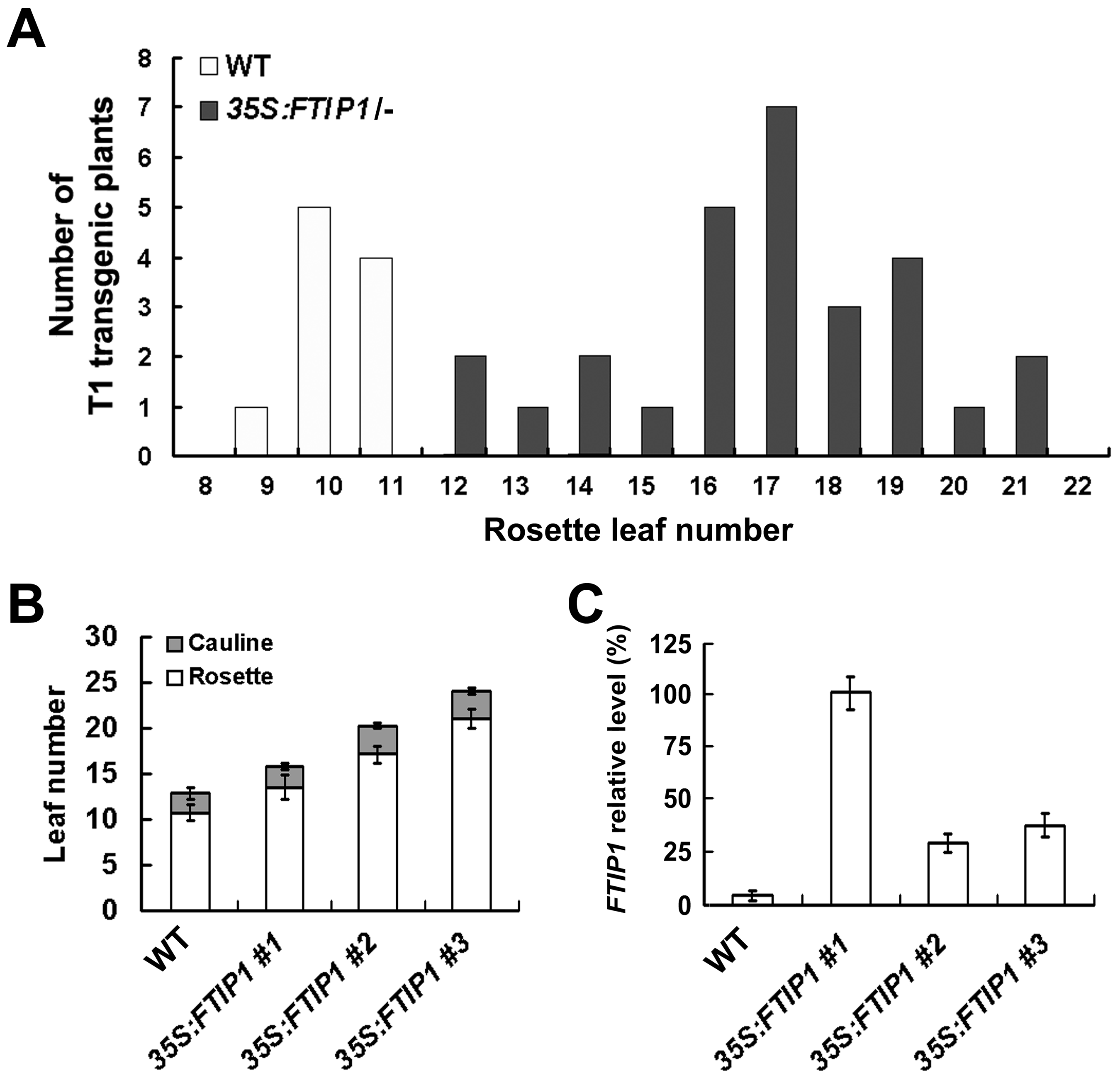

Supplement: Figure S11 — Overexpression of FTIP1 causes late flowering. (A) Distribution of flowering time in 35S:FTIP1 T1 transgenic plants. Among 28 independent lines generated, all of them show different degrees of late flowering. (B) Homozygous transgenic plants (T3 generation) of three representative 35S:FTIP1 lines consistently show late flowering. 35S:FTIP1 #1, 35S:FTIP1 #2, and 35S:FTIP1 #3 exhibit weak, moderate, and strong flowering phenotypes, respectively. Error bars indicate SD. (C) FTIP1 expression is elevated in 35S:FTIP1 lines. The degrees of late flowering in 35S:FTIP1 shown in (B) are not related to the elevated levels of FTIP1 in 35S:FTIP1 #1, 35S:FTIP1 #2, and 35S:FTIP1 #3. 9-d-old wild-type and transgenic seedlings grown under LDs were harvested for expression analysis by quantitative real-time PCR. Results were normalized against the expression of TUB2. Error bars indicate SD. (TIF) [file pbio.1001313.s011.tif]

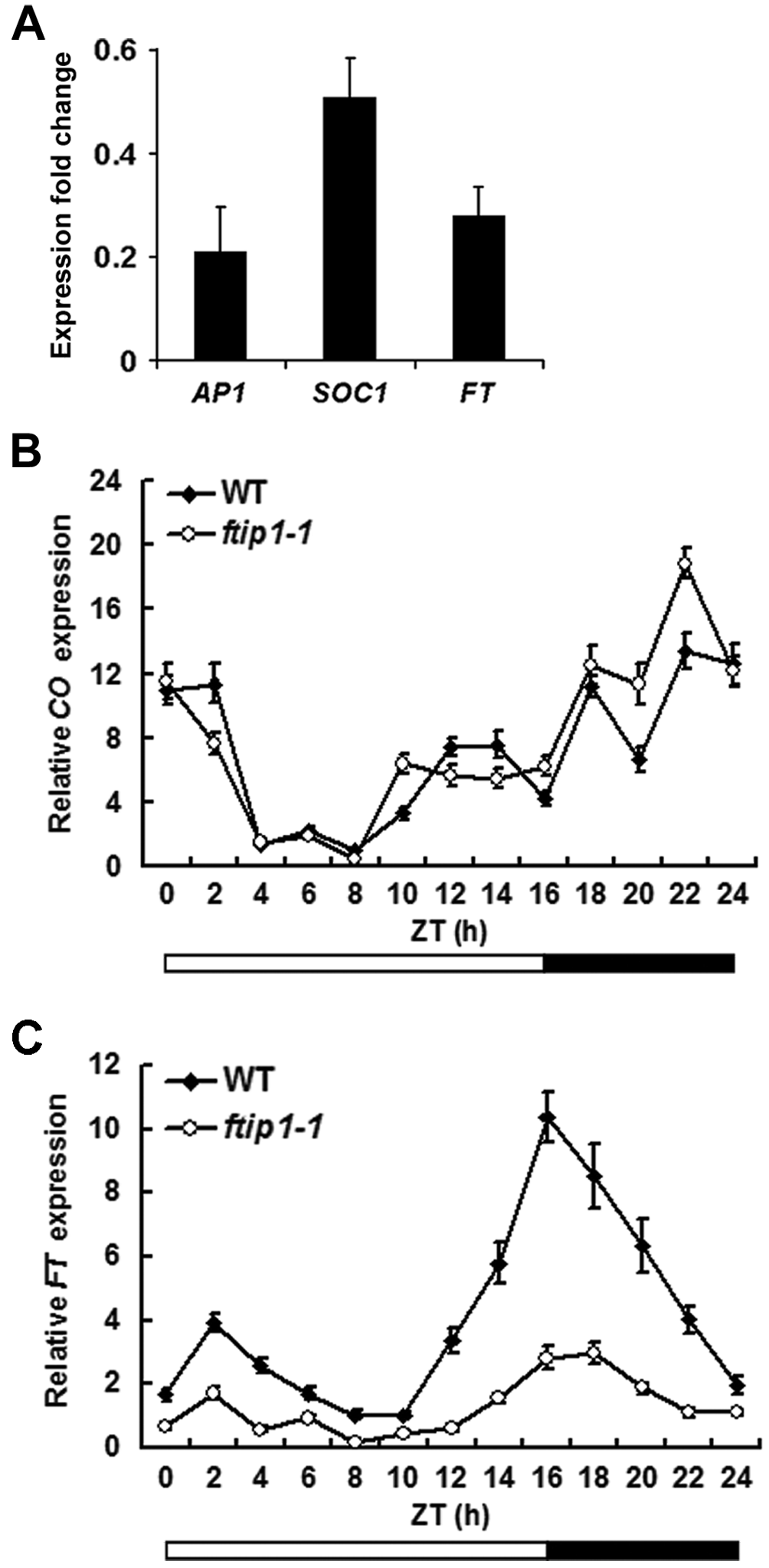

Supplement: Figure S12 — Expression of several key flowering genes in ftip1-1. (A) Expression of AP1, SOC1, and FT is downregulated in ftip1-1. 9-d-old wild-type and ftip1-1 seedlings grown under LDs were harvested for expression analysis. The gene expression in wild-type plants is set as 1. (B) CO expression is not significantly changed in ftip1-1 within a 24-h cycle under LDs. (C) FT expression is consistently downregulated in ftip1-1 within a 24-h cycle under LDs. In (B and C), 9-d-old wild-type and ftip1-1 seedlings grown under LDs were harvested at 2-h intervals over a 24-h period for expression analysis. Gene expression in (A–C) was determined by quantitative real-time PCR and normalized against TUB2 levels. Error bars indicate SD. (TIF) [file pbio.1001313.s012.tif]

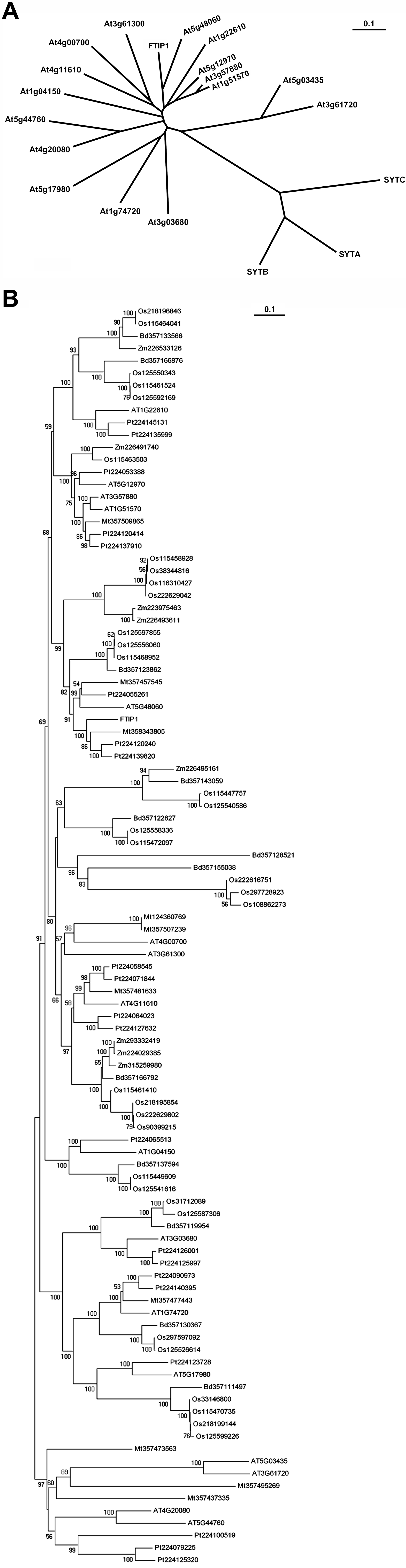

Supplement: Figure S13 — Phylogenetic analysis of FTIP1-like proteins. (A) Phylogenetic tree showing FTIP1 homologs and synaptotagmins in Arabidopsis. The phylogenetic tree was generated based on the protein alignment of FTIP1, its 16 Arabidopsis homologs, and three Arabidopsis synaptotagmins (SYTA, SYTB, and SYTC). The scale bar represents 0.1 amino acid substitution. (B) Phylogenetic analysis of FTIP1-like proteins in different plant species. The phylogenetic tree was constructed with the neighbor-joining algorithm using the program MEGA 5.05 based on the alignment of the amino acid sequences of FTIP1-like proteins. Each terminal node of the tree is labeled by the two-letter abbreviation of the corresponding species name and the unique identifier. Bootstrap values (>50%) in 500 replicates are indicated next to the nodes. Zm, Zea mays; Os, Oryza sativa; Mt, Medicago truncatula; Pt, Populus trichocarpa; Bd, Brachypodium distachyon. (TIF) [file pbio.1001313.s013.tif]
