# Supplementary material for: FTIP1 Is an Essential Regulator Required for Florigen Transport
Source: PLoS Biol. 2012 Apr 17;10(4):e1001313. doi: 10.1371/journal.pbio.1001313 (PMC3328448; doi:10.1371/journal.pbio.1001313)
Supplement: Table S1 — List of potential FT-interacting proteins isolated from the yeast two-hybrid screening. (PDF) [file pbio.1001313.s014.pdf]

Table S1. List of potential FT-interacting proteins isolated from the yeast two-hybrid screening.

Locus Identifier	Annotation
AT1G06850	AtbZIP52 (Arabidopsis thaliana basic leucine zipper 52)
AT1G20960	EMB1507 (Embryo Defective 1507)
AT1G22300	GRF10 (General regulatory factor 10)
AT1G26480	GRF12 (General regulatory factor 12)
AT5G38480	GRF3 (General regulatory factor 3)
AT1G78300	GRF2 (General regulatory factor 2)
AT1G61520	LHCA3 (Photosystem I light harvesting complex gene 3)
AT1G58100	TCP8 (TCP family transcription factor)
AT1G69690	TCP15 (TCP family transcription factor)
AT1G72010	Putative TCP family transcription factor
AT1G75240	ATHB33 (Arabidopsis thaliana homeobox protein 33)
AT3G04810	AtNek2 (NIMA-related serine/threonine kinase)
AT1G80920	J8 (DnaJ heat shock protein)
AT3G44110	ATJ3 (DnaJ heat shock protein)
AT5G06850	FTIP1 (C2 domain-containing protein)
AT5G01800	Saposin B domain-containing protein
AT4G34000	ABF3/DPBF5 (Abscisic acid responsive element-binding factor 3)
AT5G49920	Octicosapeptide/Phox/Bem1p (PB1) domain-containing protein

Genes in this list appeared at least twice in the screening.
