# Supplementary material for: FTIP1 Is an Essential Regulator Required for Florigen Transport
Source: PLoS Biol. 2012 Apr 17;10(4):e1001313. doi: 10.1371/journal.pbio.1001313 (PMC3328448; doi:10.1371/journal.pbio.1001313)
Supplement: Table S2 — Primers used for expression analyses in this study. (PDF) [file pbio.1001313.s015.pdf]

Table S2. Primers used for expression analyses in this study.

Primer Name	Sequence
	Real-time PCR
FTIP1-F	5’-CTAGTGACAGGAGCCAACCA-3’
FTIP1-R	5’-TGTTCTTCAAATGGCTCTGC-3’
TUB2-F	5’-GAGAATGCTGATGAGTGCATGG-3’
TUB2-R	5’-AGAGTTGAGTTGACCAGGGAACC-3’
AP1-F	5’- CATGGGTGGTCTGTATCAAGAAGAT-3’
AP1-R	5’- CATGCGGCGAAGCAGCCAAGGTT-3’
SOC1-F	5’-AGCTGCAGAAAACGAGAAGCTCTCTG-3’
SOC1-R	5’-GGGCTACTCTCTTCATCACCTCTTCC-3’
FT-F	5’-CTTGGCAGGCAAACAGTGTATGCAC-3’
FT-R	5’-GCCACTCTCCCTCTGACAATTGTAGA-3’
FT(UTR)-F	5’-GAAGACTTTAGATGGCTTCTTCC-3’
FT(UTR)-R	5’-GAACTACTATAGGCATCATCACC-3’
CO-F	5’-TCAGGGACTCACTACAACGACAATGG-3’
CO-R	5’-TTGGGTGTGAAGCTGTTGTGACACAT-3’
GFP-F1	5’-GACTTCTTCAAGAGCGCCAT-3’
GFP-R1	5’-CCCTTAAGCTCGATCCTGTT-3’
	Semi-quantitative PCR
FTIP1-F	5’- ATGGCAGCCAAAGATGGAGC -3’
FTIP1-R	5’- TGTTCTTCAAATGGCTCTGC -3’
TUB2-F	5’- ATCCGTGAAGAGTACCCAGAT -3’
TUB2-R	5’- TCACCTTCTTCATCCGCAGTT -3’
